# Competitive Adsorption of a Monoclonal Antibody and Nonionic Surfactant at the PDMS/Water Interface

**DOI:** 10.1021/acs.molpharmaceut.2c01099

**Published:** 2023-04-04

**Authors:** Kangcheng Shen, Xuzhi Hu, Zongyi Li, Mingrui Liao, Zeyuan Zhuang, Sean Ruane, Ziwei Wang, Peixun Li, Samantha Micciulla, Narayanan Kasinathan, Cavan Kalonia, Jian Ren Lu

**Affiliations:** † Biological Physics Laboratory, Department of Physics and Astronomy, University of Manchester, Oxford Road, Schuster Building, Manchester M13 9PL, U.K.; ‡ National Graphene Institute, University of Manchester, Oxford Road, Schuster Building, Manchester M13 9PL, U.K.; § STFC ISIS Facility, Rutherford Appleton Laboratory, Didcot OX11 0QX, U.K.; ∥ Institut Laue Langevin, 71 Avenue des Martyrs, CS-20156, Grenoble 38042, France; ⊥ Dosage Form Design & Development, BioPharmaceutical Development, BioPharmaceuticals R&D, AstraZeneca, Cambridge CB21 6GH, U.K.; # Dosage Form Design & Development, BioPharmaceutical Development, BioPharmaceuticals R&D, AstraZeneca, Gaithersburg, Maryland 20878, United States

**Keywords:** mAb, interfacial adsorption, polysorbate 80, neutron reflection, polydimethylsiloxane, spectroscopic ellipsometry, ToC

## Abstract

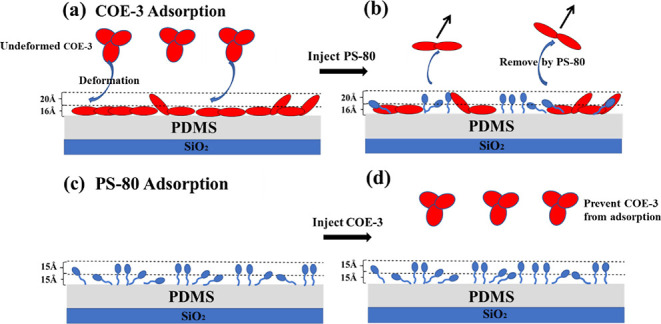

Interfacial adsorption of monoclonal antibodies (mAbs) can cause structural deformation and induce undesired aggregation and precipitation. Nonionic surfactants are often added to reduce interfacial adsorption of mAbs which may occur during manufacturing, storage, and/or administration. As mAbs are commonly manufactured into ready-to-use syringes coated with silicone oil to improve lubrication, it is important to understand how an mAb, nonionic surfactant, and silicone oil interact at the oil/water interface. In this work, we have coated a polydimethylsiloxane (PDMS) nanofilm onto an optically flat silicon substrate to facilitate the measurements of adsorption of a model mAb, COE-3, and a commercial nonionic surfactant, polysorbate 80 (PS-80), at the siliconized PDMS/water interface using spectroscopic ellipsometry and neutron reflection. Compared to the uncoated SiO_2_ surface (mimicking glass), COE-3 adsorption to the PDMS surface was substantially reduced, and the adsorbed layer was characterized by the dense but thin inner layer of 16 Å and an outer diffuse layer of 20 Å, indicating structural deformation. When PS-80 was exposed to the pre-adsorbed COE-3 surface, it removed 60 wt % of COE-3 and formed a co-adsorbed layer with a similar total thickness of 36 Å. When PS-80 was injected first or as a mixture with COE-3, it completely prevented COE-3 adsorption. These findings reveal the hydrophobic nature of the PDMS surface and confirm the inhibitory role of the nonionic surfactant in preventing COE-3 adsorption at the PDMS/water interface.

## 1 Introduction

Recent advances in biomedical science and engineering have led to a surge of interest in developing therapeutic monoclonal antibodies (mAbs) as the systemic treatments for major human diseases such as cancers, infections, autoimmune diseases, and Alzheimer’s disease.^1−3^ During the COVID-19 pandemic outbreak, several companies have developed prophylactic or therapeutic mAbs which target the spike protein of the Sars-Cov-2 virus. These mAbs were ready for clinical evaluation and manufacturing within months.^4^ The fast development of mAb biopharmaceuticals has initiated active research endeavors, many of which are focused on predicting mAb degradation. Immunoglobulin G (IgG) is the most common type of mAb therapeutic, bearing the structural hallmark of 2 Fabs and 1 Fc. Like most proteins, these mAbs are amphiphilic with high affinity to adsorb at accessible interfaces, potentially causing structural deformation and unfolding. The desorption of partially deformed mAbs could induce further structural unfolding of other mAbs through aggregation, leading to precipitation.^1^

Interfacial adsorption is widely regarded as a crucial process potentially mediating mAb subvisible particle formation during manufacturing, storage, transport, and/or administration.^5,6^ Dosages of mAbs administered via intramuscular and subcutaneous injection are commonly stored in pre-filled syringes^7^ and are formulated with a nonionic surfactant.^8,9^ The pre-filled syringes are often lubricated with silicone oil to reduce friction and achieve smooth injection.^7^ To mitigate mAb aggregation and improve the stability of the formulated product, nonionic surfactants such as polysorbate 80 (PS-80) are commonly used to reduce mAb surface adsorption.^10−13^ In spite of extensive studies,^14−17^ the availability of detailed structural information about how an mAb and surfactant adsorb on the oil film is limited. A recent study indicated that mAbs and nonionic surfactants might co-adsorb at the oil/water interface.^17^ It is useful to understand how mAbs and surfactants adsorb alone^18^ and as a mixture at the silicone oil/water interface and how the interfacial adsorption processes impact the structure of the adsorbed mAb molecules. This insight could help optimize formulation design and/or surface treatments.

Recent studies have demonstrated that an mAb such as COE-3 and its constituent fragments, antigen-binding fragment (Fab) and fragment crystallization (Fc), can all adsorb at the hexadecane/water interface.^14^ Although interfacial tension measurements can provide information on protein adsorption, little information on the adsorbed amount or structural changes of the protein can be gleaned because application of the Gibbs equation is limited by the non-equilibrating nature of the system and deviation from non-ideality.^19^ Neutron reflection (NR) in combination with deuterium labeling is capable of unraveling the thickness and composition of the interfacial layer from which information about the physical state of the adsorbed mAb can be inferred, but a stable oil film of a few μm must be formed on the optically flat substrate such as silicon to facilitate NR with sufficient beam transmission. The instability of the oil layer against the surfactant makes it challenging to examine the quantity, orientation, and structure of the adsorbed protein molecules.^14^

Silicone oil is widely used in medical and pharmaceutical sectors because of its good versatility and biocompatibility.^20^ Generally, “bake on” and “spray on” are 2 common types of siliconization applied to syringes, while manufacturers prefer “bake on” to minimize the silicon oil migration into the product.^21^ In this work, a thin polydimethylsiloxane (PDMS) film is prepared on a SiO_2_ surface and heated to anneal the “bake on” silicon film. The PDMS oil film is first coated onto an optically flat and well characterized silicon substrate by spin coating and then cross-linked onto the native SiO_2_ surface via thermal annealing, providing a better oil film stability. Similar silanized PDMS nanofilms on SiO_2_ surfaces have been used to study the adsorption behavior of mAb–surfactant mixtures using techniques such as quartz crystal microbalance with dissipation.^15,16^ The structural features of the adsorbed surfactant–mAb complexes at the interface remain poorly characterized due to the lack of resolution in distinguishing different components in the mixed system.

In this work, we demonstrate that NR together with deuterium labeling to nonionic surfactants provides appropriate resolution in revealing the structural details through parallel NR measurements under different isotopic constrasts.^22,23^ This approach is particularly effective at unraveling the thickness and composition of the mixed mAb–surfactant layer from which changes in the physical state of the adsorbed mAb molecules and their relative location to the surfactant could be determined.^13,24^

An isotype of the human IgG1κ antibody, COE-3,^11^ was used as the model mAb to investigate interfacial adsorption behavior in the presence of PS-80. The dynamic adsorption was followed by spectroscopic ellipsometry (SE). To aid the assessment of interfacial molecular interactions and highlight technical implications, the process was conducted in 3 different sequential injections, COE-3 followed by PS-80, PS-80 followed by COE-3, and the pre-mixed solution of COE-3 and PS-80. Where co-adsorption occurred, NR measurements under different contrasts helped unravel the internal composition and structure. Thus, the combined SE and NR experiments helped us follow how the adsorption of COE-3 onto PDMS is mediated by PS-80. This work provides new insights into the complex interactions between mAbs and surfactants and also offers useful indications about how a surfactant is used in mediating mAb adsorption in formulation design.

## 2 Experimental Section

### 2.1 Materials

COE-3, provided by AstraZeneca (Cambridge, UK), has a molecular weight of 144,560 Da and an isoelectric point of 8.44.^24^ The stock of COE-3 was dissolved in histidine/histidine hydrochloride buffer (25 mM, 7% w/v sucrose, pH 5.5) denoted His buffer. The COE-3 stock solution was then diluted directly under the same buffer without sucrose. The effect of sucrose is regarded as negligible since the sucrose concentration is diluted to around 0.01% w/v. The Fc and Fab were generated by papain digestion of COE-3. The stocks of Fc and Fab were supplied at the concentrations of 55.6 and 45.0 mg/mL and their isoelectric points of 6.36 and 9.64, respectively.^25^ In the papain digestion process, as Fc and Fab solutions are dialyzed many times to get separated from each other, the effect of sucrose solution can be regarded as negligible as well. PS-80, also denoted h-PS-80 (20 ethoxylates, molecular weight: 1310 g/mol, and density: 1.102 g/cm^3^), was purchased from Fisher BioReagents (99% purity) and used without further purification unless otherwise stated. The ethoxylate-deuterated PS-80, denoted d-PS-80, was synthesized in the ISIS Deuteration Laboratory (98% D). The PS-80 samples were dissolved in His buffer at a concentration of 200 ppm, a typical concentration for industrial application, which is much higher than its critical micellar concentration (CMC). In this experiment, the CMC values of PS-80 and head-deuterated PS-80 were found to be 16 and 17 ppm, respectively. The results were obtained from the surface tension measurements at the air/liquid interface using Krüss tensiometer BP100, as shown in Figure S1, and were in agreement with the reported value 0.013 mM.^10^

### 2.2 Surface Modification by PDMS

Silicon wafers (⟨111⟩, 4 inch diameter, with one side polished, from PI-KEM) were cut into 1 cm × 1 cm × 1 mm pieces for SE measurements. Silicon blocks (also ⟨111⟩) with dimensions of 5 cm × 8 cm × 1.2 cm were used for NR measurements. Before surface modification, the silicon wafers and blocks were cleaned by a mixture of 90 v % H_2_SO_4_ (98%) and 10 v % H_2_O_2_ (30%) at 90 °C for 3 min. PDMS (vinyl-terminated, average molecular weight 25,000 g/mol, density 0.965 g/cm^3^, viscosity 850–1150 cSt at 25 °C, Sigma-Aldrich) was first dissolved in hexane at designated concentrations. 100 μL of PDMS solution (or 1.5 mL for a Si block) was dropped onto a clean silicon wafer, followed by spin-coating at 3000 rpm for 30 s. The coated wafers/blocks were annealed at 150 °C for 8 h to improve the stability of the coated PDMS films against buffer rinsing or surfactant attack. Before any measurement, the PDMS-modified silicon wafers/blocks were rinsed with plenty of deionized water to remove any residues.

### 2.3 Contact Angle Measurements and AFM Imaging

The advancing contact angle was measured by a drop shape analyzer KRÜSS DSA25S. An aliquot (2 μL) of deionized water was dropped onto the PDMS film surface. The contact angle was determined by curve fitting.

The atomic force microscopy (AFM) characterization of the PDMS film surface in air was performed on a Bruker Multimode 8 force microscope. The scans were carried out in the SCANASYST mode using a silicon probe on a nitride cantilever in air (model: scanasyst-air, Bruker Ltd.). The AFM characterization of the PDMS film surface under water was performed in the SCANASYST liquid mode on a Bruker Catalyst using a scanasyst-fluid + probe.

### 2.4 Spectroscopic Ellipsometry

SE measurements were performed in a specific liquid cell with a fixed incident angle of 70° at the solid/liquid interface using a Woollam spectroscopic ellipsometer (J.A. Woollam Co. Inc.).^27^ Three sequential injections as stated above were studied. For the first set, COE-3 was first injected at 10, 100, and 1000 ppm. When protein adsorption reached a plateau, each COE-3 solution was removed, followed by PS-80 injection at a surfactant concentration of 200 ppm, which is 13 times than its CMC. For the second set, the injection was reversed, starting with 200 ppm PS-80 injection, followed by 100 ppm COE-3 injection. For the third set, 100 ppm COE-3 and 200 ppm PS-80 were first mixed and then injected into the cell. The dynamic measurements of COE-3 and surfactant adsorption processes were undertaken under the “in situ” mode, and the adsorbed amount was calculated using the De Feijter’s equation,^28^ with a detailed description of SE measurements presented in Section S1 of the Supporting Information. Because the PDMS films were only a few nm thick, SE was incapable of unraveling their thickness from composition. In the SE data analysis, the densities of the PDMS films were kept the same as those of the bulk PDMS fluid. The thicknesses obtained were thus nominal.

### 2.5 Neutron Reflection

NR experiments were performed on SURF and D17 reflectometers^29^ using the same setup as previously reported^30^ at ISIS, Didcot, UK, and Institut Laue-Langevin, Grenoble, France, respectively. The silicon blocks modified by PDMS coating were clamped against a liquid cell to facilitate the solid/liquid measurements. The PDMS layer was first characterized in 3 contrasts: D_2_O, contrast-matched air (CMAir, D_2_O/H_2_O = 0.081:0.919), and contrast-matched COE-3 (CMCOE-3, D_2_O/H_2_O = 0.454:0.546). All scattering length densities (SLDs) of the materials used in this experiment are listed in Table S1. As COE-3 has labile hydrogens associated with polar groups such as −NH_2_, −OH, and −NH–, which will undertake H/D exchanges with the bulk solvent, COE-3 has different SLDs in different contrast solutions. The measurements of COE-3 and PS-80 adsorption were undertaken in the 3 different sequential injections, COE-3 followed by PS-80, PS-80 followed by COE-3, and the pre-mixed solution of COE-3 and PS-80, with each measurement performed under the same three contrasts for 1 h or more depending on the specific isotopic contrast.

The reflectivity data were analyzed using Motofit software,^31^ and the multiple layer model was used to fit COE-3/PS-80 co-adsorption at the PDMS/water interface, with the detailed NR principles given in Section S2 of the Supporting Information.

## 3 Results

### 3.1 PDMS Nanofilm Coating and Characterization

The thickness of the native silicon oxide layer on a silicon wafer or block was first measured by SE. The thicknesses of the SiO_2_ layers were 14 ± 3 Å. The thicknesses of the coated PDMS layers showed an almost linear dependence on the PDMS concentration, and annealing caused a slight decrease of the thickness, as shown in Figure S2. As explained already, these thicknesses were obtained by keeping the densities of the films the same as those of the bulk PDMS during SE data fitting to the measured amplitude Ψ and phase difference Δ. The contact angle increased from below 10° at the bare oxide surface to over 95° upon siliconization, as shown in Figure S3, indicating that the surface was transformed from hydrophilic to hydrophobic. AFM imaging (Figure S4) revealed the smoothly cured PDMS films across the entire scanned PDMS film surfaces with a roughness of <0.5 nm. No obvious morphological changes were observed under exposure to the His buffer solution. Changes in film thickness from different coating PDMS solutions did not appear to alter these features. As both SE and NR measurements benefit from a smooth and thin film coated on the solid substrate, 0.05 wt % PDMS solution was then chosen for film coating. Representative SE results are shown in Figure S6 and were measured at 0, 15, and 30 min of buffer immersion. Small changes in Ψ and Δ were observed, indicating that the PDMS films were stable, and the film surfaces were sharp and smooth at the PDMS/water interface.

The structural features of the PDMS films coated from the same PDMS solution were further characterized by NR under 3 water contrasts as described above. Simultaneous fitting to the reflectivity profiles (Figure S7) measured revealed that the PDMS films immersed in the buffer had thicknesses of 50 ± 5 Å, consistent with SE measurements. The best fitting results are shown in Table S2. The volume fraction of PDMS in these films was over 90%, indicating that the PDMS films were dense but contained some hydration or very small surface defects, consistent with AFM imaging. These characterizations showed good consistency when compared to a good “bake on” PDMS model with a thickness of 10 nm.^21^

### 3.2 Interfacial Adsorption of PS-80 and COE-3

#### 3.2.1 COE-3 Adsorption at the PDMS/Water Interface

**Figure 1:**
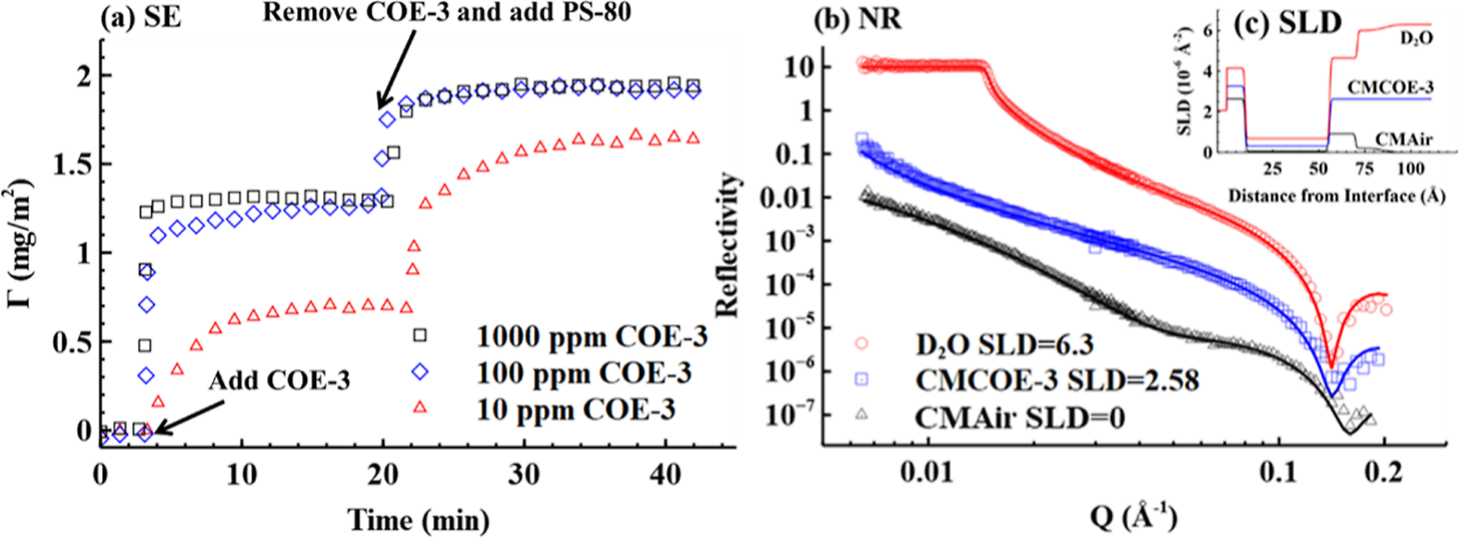
(a) Adsorption of COE-3 at concentrations of 10, 100, and 1000 ppm under pH 5.5 (25 mM His buffer) on the PDMS surface, followed by the removal of COE-3 solution and then the injection of PS-80 at 200 ppm in the same buffer. (b) NR profiles measured at the PDMS/water interface for COE-3 adsorption at a concentration of 100 ppm in D_2_O, CMCOE-3, and CMAir under pH 5.5 (25 mM His buffer). For better visualization, the profiles are multiplied by 10, 1, and 0.1 from top to bottom. The fitted SLD profiles for the NR results are shown in (c).

Adsorption of COE-3 at concentrations of 10, 100, and 1000 ppm at the PDMS/water interface was first examined using SE. The measurements were repeated three times for each mAb concentration, and the results were highly reproducible. The stability of the coated PDMS film was first checked by rinsing with His buffer during the time-dependent run, and negligible fluctuations were observed, indicating that the PDMS film was stable against buffer rinse without any time-dependent change. COE-3 samples at different concentrations were then measured. Figure 1a shows the exemplar time-dependent adsorption of COE-3 onto the PDMS surface. The adsorption of COE-3 at the two higher concentrations (100 and 1000 ppm) showed fast adsorption and reached a plateau within the first 1 min, while the equilibrating time for COE-3 at a low concentration of 10 ppm was about 10 min.

The adsorbed amount can be obtained from eq S3 based on the SE measurements. The plateau amount of the adsorbed COE-3 at 10 ppm was found to be 0.70 ± 0.05 mg/m^2^. In contrast, the values at 100 and 1000 ppm were higher, at 1.27 ± 0.05 and 1.31 ± 0.05 mg/m^2^, respectively. These results indicate that COE-3 reached saturated adsorption at the concentration around 100 ppm. The SE results of the 40,000 ppm COE-3 adsorption are illustrated in Figure S5. The SE results reveal that even at a concentration of 40,000 ppm, the COE-3 adsorbed amount is still around 1.3 mg/m^2^. By comparing SE measurements before and after buffer rinsing, it can be observed that the adsorbed COE-3 molecules could not be desorbed, indicating that the adsorption of COE-3 at the PDMS/water interface is irreversible under the conditions evaluated.

The structural features of the adsorbed COE-3 molecules at the PDMS/water interface were subsequently determined using NR. As COE-3 adsorption became saturated at 100 ppm, the NR experiments were carried out at 100 ppm of COE-3 under the 3 isotropic contrasts of D_2_O, CMCOE-3, and CMAir. Representative profiles together with the best fits and corresponding SLD profiles are shown in Figure 1b,c, respectively. The SLD changes of silicon oxide and PDMS are caused by hydration of different contrast solutions. A two-layer model was used to fit the adsorbed COE-3 layer with each layer being composed of COE-3 and water, as described in Section S2 in the Supporting Information. The best fit parameters are shown in Table S3. The volume fraction and adsorbed amount in each layer can be calculated from eqs S5 and S6, and the values are listed in Table 1. No penetration of COE-3 into the PDMS layer was observed. From the PDMS substrate to the bulk solvent, layers 1 and 2 have thicknesses of 16 ± 2 and 20 ± 2 Å, and the volume fraction for COE-3 is 0.5 and 0.1, respectively. Dense layer 1 and diffuse layer 2 suggest that COE-3 deformed on the PDMS surface since the shortest axial length of COE-3 is 40–45 Å,^26^ which is much larger than that of layer 1. Diffuse layer 2 has a volume fraction of 0.1, suggesting the existence of a small number of tilted projections from Fc or Fab fragments. However, due to the limitation of the experiment resolution, the actual domain conformation cannot be determined. Compared to the dimensions of COE-3 (45 × 90 × 180 Å), the most likely conformation of the adsorbed layer is “flat on” with deformation.^26^ The structural conformations of the adsorbed COE-3 molecules are schematically shown in Figure 4a. The undeformed COE-3 in bulk is represented with elliptical domains, while the adsorbed COE-3 is represented with deformed elliptical domains. The adsorbed amounts of COE-3 in layers 1 and 2 are at 1.21 ± 0.11 and 0.28 ± 0.05 mg/m^2^, respectively. Thus, the total adsorbed COE-3 as calculated by NR is 1.49 ± 0.12 mg/m^2^, consistent with the value of 1.25 ± 0.1 mg/m^2^ measured by SE within error. As the main body of the COE-3 molecule must be accommodated in dense layer 1 with a thickness less than half of the shortest axial length, molecular deformation could result in a large number of direct contacts with the PDMS surface. Such deformation and lateral overlapping must be responsible for the irreversible adsorption.

**Table 1:** Best Fit Structural Parameters to Neutron Reflectivity Profiles Measured Following COE-3 Adsorption (100 ppm, at pH 5.5, 25 mM Histidine Buffer) at the PDMS/Water Interface Using a Two-Layer Model to Describe the COE-3 Distribution^a^

layer	thickness (Å)	Φ_COE-3_	Φ_water_	Γ_COE-3_ (mg/m^–2^)
PDMS	50 ± 5	0	0.1	0
1	16 ± 2	0.5 ± 0.05	0.5 ± 0.05	1.21 ± 0.11
2	20 ± 2	0.1 ± 0.02	0.9 ± 0.05	0.28 ± 0.05

a Φ denotes the volume fraction, and Γ denotes the adsorbed amount per unit area.

#### 3.2.2 Adsorption of Fc and Fab on the PDMS Layer

**Figure 2:**
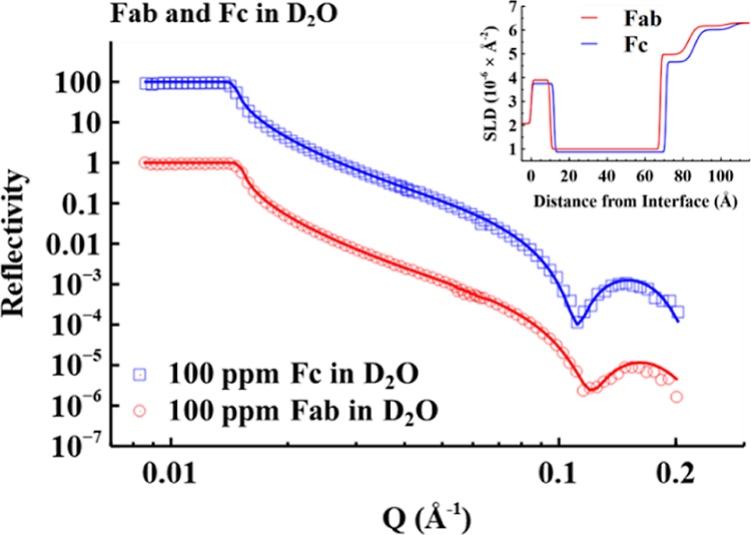
NR profiles for the surface adsorption of Fc and Fab fragments at 100 ppm, pH 5.5 and 25 mM histidine buffer. For better visualization, the profile of Fc is multiplied by 100. The SLD profiles are illustrated in the top right inset.

The adsorption behavior of Fc and Fab fragments of COE-3 was also characterized by NR at a concentration of 100 ppm (pH 5.5, 25 mM histidine buffer) in D_2_O solution. The representative NR profiles and the best fits are illustrated in Figure 2. A two-layer model was applied to fit the adsorbed Fc and Fab layers, and the best fitting parameters are shown in Table S4. The structural details of the adsorbed Fc and Fab layers were again revealed by solving eqs S5 and S6, and the best fitted results are listed in Table 2. Both Fc and Fab formed a 16 Å dense layer and a 20 Å diffuse layer with the adsorbed amounts of 1.36 and 1.14 mg/m^2^, respectively. Since the short lengths of Fab or Fc are equal to the minimum axial length of COE-3 (40–45 Å), both Fc and Fab parts must be deformed on the PDMS layer. The diffuse layers of Fc and Fab may indicate the coexistence of some tilted Fc and Fab fragments. The higher adsorbed amount of Fc arises from its relative high hydrophobicity,^25^ which induces greater adsorption on the hydrophobic PDMS surface. Thus, the adsorbed layer structures are similar among Fab, Fc, and the whole COE-3 molecules.

**Table 2:** Fitted Structural Parameters of the Neutron Profiles Illustrated in Figure 2^a^

material	layer	thickness (Å)	Φ_Fc_	Φ_water_	Γ_Fc_ (mg/m^2^)
Fc	PDMS	59 ± 5	0	0.13	0
	1	16 ± 2	0.46 ± 0.05	0.54 ± 0.05	1.09 ± 0.11
	2	20 ± 2	0.10 ± 0.02	0.90 ± 0.05	0.27 ± 0.05

material	layer	thickness (Å)	Φ_Fab_	Φ_water_	Γ_Fab_ (mg/m^2^)
Fab	PDMS	58 ± 5	0	0.15	0
	1	16 ± 2	0.43 ± 0.05	0.57 ± 0.05	0.95 ± 0.11
	2	20 ± 2	0.07 ± 0.02	0.93 ± 0.05	0.19 ± 0.05

a The best fitted parameters are listed in Table S4.

#### 3.2.3 Adsorption of PS-80 onto the Pre-Adsorbed COE-3 Layer

**Figure 3:**
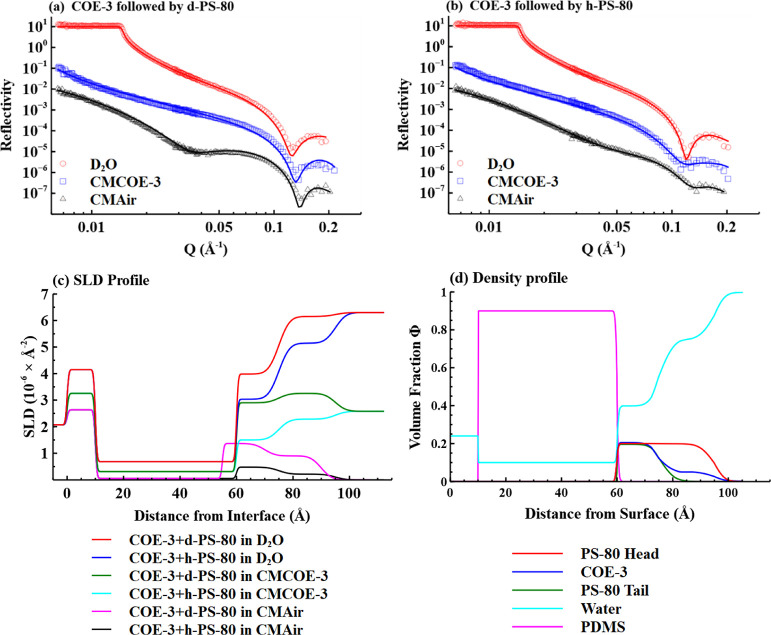
NR profiles for the sequential adsorption starting from COE-3 at 100 ppm, pH 5.5 in 25 mM His buffer followed by PS-80 at 200 ppm, pH 5.5 in 25 mM His buffer including (a) head-deuterated PS-80 and (b) protonated PS-80. For better visualization, the profiles are multiplied by 10, 1, and 0.1 from top to bottom. (c,d) show the SLD and density profiles obtained from the best fitting results, respectively.

After the completion of COE-3 adsorption, the mAb solution was removed, followed by His buffer rinsing. Upon rinsing, SE revealed no change in the adsorbed amount, which is consistent with the result of irreversible adsorption. PS-80 solution at 200 ppm (about 15 times above CMC, under the same His buffer at pH 5.5 and an ionic strength of 25 mM) was subsequently injected onto the pre-adsorbed COE-3 layer, and the co-adsorption was also determined. Figure 1a shows the dynamic adsorption of PS-80 onto the COE-3 layers pre-adsorbed at the concentrations of 10, 100, and 1000 ppm. Rapid increments for the total adsorbed amount were observed following all 3 PS-80 injections. For the PS-80 adsorbed onto the COE-3 layers at the two high protein concentrations of 100 and 1000 ppm, adsorption reached a plateau within 1 min, with the total adsorbed amount increasing to around 2 mg/m^2^. In contrast, the total amount of PS-80 adsorbed onto the COE-3 layer pre-adsorbed from 10 ppm mAb solution increased to 1.2 mg/m^2^ in 1 min, and the subsequent equilibrating process led to the total final amount of 1.6 mg/m^2^ after 10 min. The increase in the total adsorbed amount upon PS-80 injection was then attributed to the adsorption of the surfactant. While a net increase of about 1 mg/m^2^ of PS-80 was adsorbed onto the 10 ppm pre-adsorbed COE-3 layer, only 0.6 mg/m^2^ of PS-80 was adsorbed onto the 100 and 1000 ppm pre-adsorbed COE-3 layers. The higher adsorbed amount of PS-80 on the 10 ppm pre-adsorbed COE-3 layer must indicate that PS-80 was adsorbed on the unoccupied PDMS area since the COE-3 adsorption was unsaturated. However, the results of the adsorption of PS-80 were obtained under the assumption that there was no exchange between PS-80 and COE-3, which cannot be verified using SE. To unravel the structure across the adsorbed interfacial layers, NR measurements were undertaken with deuterium labeling to the ethoxylate head of PS-80. The isotopic labeling helped highlight the location of the surfactant within the mixed interfacial film. The NR experiments were undertaken under 6 contrasts (h/d-PS-80 in D_2_O, CMCOE-3, and CMAir). The reflectivity profiles for d-PS-80 adsorption onto the pre-adsorbed COE-3 in the 3 solvent contrasts along with the best fits are shown in Figure 3a and those for h-PS-80 co-adsorption in Figure 3b. To optimize the best fits to all 6 contrasts, a two-layer model was found to be appropriate with each layer composed of COE-3, water, and the PS-80 head and tail. As described in Section S2 in the Supporting Information, all six contrasts were simultaneously fitted to the best optimized SLD of each layer. The layer average SLD equals the sum of the volume fraction of each component multiplied by its SLD, as indicated by eq S5. As the layer average SLD was fitted and SLD for each component was calculated, as listed in Table S1, the volume fraction of each component in the layer was determined. The best fitted parameters are listed in Table S5. Figure 3c,d shows the SLD and volume fraction profiles of the components, respectively, across the mAb layers obtained from the NR model fitting, with the best-fitted parameters listed in Table 3.

**Table 3:** Best Fitted Parameters from the NR Results of PS-80 Co-Adsorption onto the Pre-Adsorbed COE-3 Layer Using a Two-Layer Model

layer	thickness (Å)	Φ_COE-3_	Φ_PS-80 tail_	Φ_PS-80 head_	Φ_water_	Γ_COE-3_ (mg/m^2^)	Γ_PS-80_ (mg/m^2^)
1	16 ± 2	0.2 ± 0.05	0.2 ± 0.05	0.2 ± 0.05	0.4 ± 0.1	0.49 ± 0.05	0.69 ± 0.05
2	20 ± 2	0.05 ± 0.02	0	0.2 ± 0.05	0.75 ± 0.1	0.14 ± 0.05	0.45 ± 0.05
total	36 ± 3					0.63 ± 0.1	1.14 ± 0.1

Substantial differences in SLD between each solvent contrast pair containing h-PS-80 and d-PS-80 can be observed from Figure 3c, indicating the presence of the surfactant across both layers 1 and 2. There was no indication from the data analysis to suggest penetration of the surfactant or protein into the PDMS film. The good fits to all reflectivity profiles and their consistency support the absence of these surface-active species in the PDMS substrate. Although the combined analysis offers a comprehensive picture about the distributions of individual components across the interface, each isotopic contrast offers different structural sensitivity. Under the CMCOE-3 contrast, COE-3 is invisible to neutrons because its SLD is equal to that of the bulk solvent, so the reflectivity is only sensitive to the surfactant distribution. Given that the SLD of CMCOE-3 is 2.58 × 10^–6^ Å^–2^, that of the surfactant tail is −0.39 × 10^–6^ Å^–2^, and that of the deuterated head is 7 × 10^–6^ Å^–2^, the average SLD of layer 1 is fitted to be 3.2 × 10^–6^ Å^–2^, implying the accumulation of the d-head of PS-80 in it. In contrast, the reflectivity under the CMAir contrast contains information about both the COE-3 and surfactant but is most sensitive to the deuterated head of PS-80 because of the largest SLD variation. Furthermore, there is a distinct variation from the SLD profiles containing d-PS-80 and h-PS-80 under the CMAir contrast, with the SLDs of layer 1 being 1.4 and 0.4 × 10^–6^ Å^–2^, respectively, also confirming the abundant gathering of the surfactant heads in layer 1.

**Figure 4:**
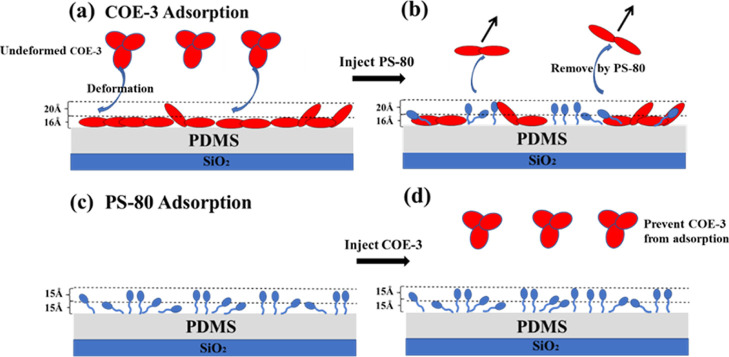
Schematic depictions of different adsorption sequences of COE-3 and PS-80 at the PDMS/water interface. (a) Adsorption of COE-3 on the PDMS layer and COE-3 was deformed at the interface; (b) injection of PS-80 on the adsorbed COE-3 layer, resulting in the removal of about 60 w % of COE-3 by PS-80; (c) adsorption of PS-80 on the PDMS layer; and (d) adsorption of COE-3 after PS-80 pre-adsorption or both co-adsorbed from a binary mixture, resulting in the complete inhibition of COE-3 adsorption.

From the changes of SLD profiles in each layer, the volume fraction of each component could be calculated, with the resultant volume fraction profiles shown in Figure 3d. As shown in Table 3, dense layer 1 has a thickness of 16 Å, containing the deformed COE-3 and the head and tail parts of PS-80. Layer 2 has a thickness of 20 Å, containing both the diffuse layer of COE-3, as described above, and the sorbitan head of PS-80. The adsorbed amount of COE-3 (Γ_COE-3_) was reduced from 1.49 ± 0.12 to 0.63 ± 0.1 mg/m^2^ upon the injection of PS-80, indicating that 60 w % of the pre-adsorbed COE-3 was removed by PS-80 co-adsorption. The co-adsorbed amount of PS-80 was calculated to be 1.14 ± 0.1 mg/m^2^. The total adsorbed amount of the protein and surfactant was estimated to be 1.77 ± 0.2 mg/m^2^ by NR and 1.91 ± 0.1 mg/m^2^ by SE, showing good consistency within error. These results together suggest that PS-80 effectively replaced some of the loosely adsorbed COE-3 molecules, occupying the vacant PDMS surface spots. The structure of the PS-80-COE-3 co-adsorbed layer on the PDMS surface is schematically shown in Figure 4b where it can be seen that the distribution of head groups of the surfactant molecules overlaps with the entire COE-3 distribution and that the alkyl chain distribution is confined to the dense inner layer.

### 3.3 Adsorption of COE-3 onto the Pre-Adsorbed PS-80 Layer

#### 3.3.1 PS-80 Adsorption at the PDMS/Water Interface

**Figure 5:**
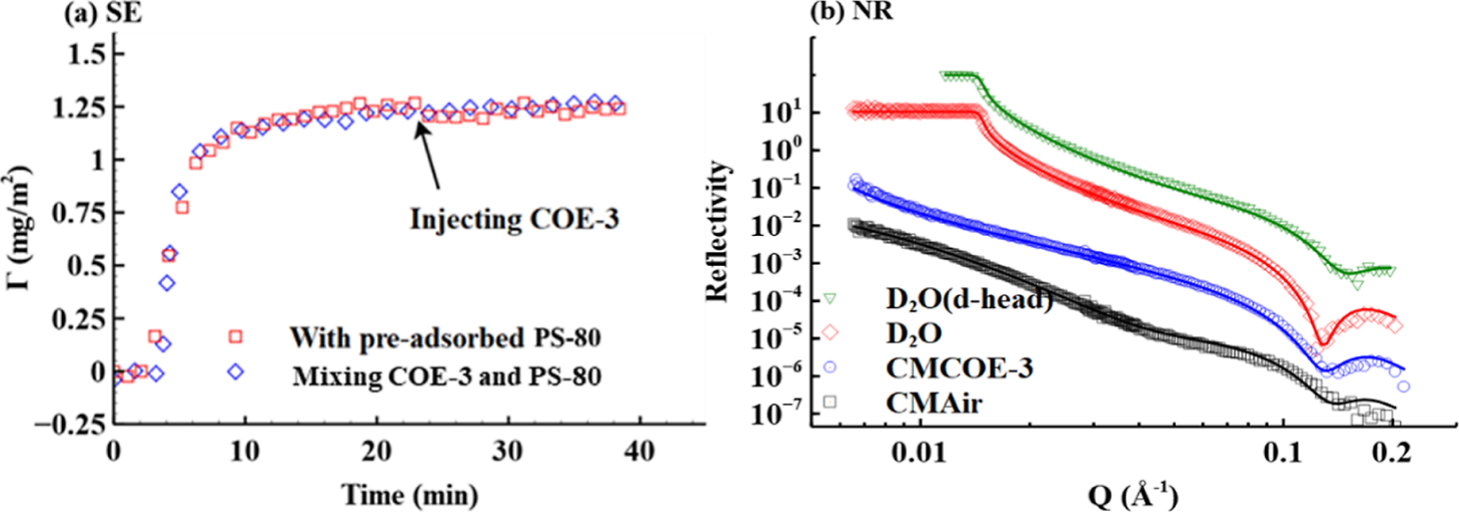
(a) SE measurement of dynamic adsorption starting from PS-80 followed by COE-3 co-adsorption at the PDMS/solution interface (red). The SE profile of the co-adsorption from the pre-mixed binary mixture of COE-3 and PS-80 is also shown (blue). (b) NR profiles measured from h-PS-80 adsorption in D_2_O, CMCOE-3, and CMAir and d-PS-80 in D_2_O with the best fits shown as continuous lines. For better visualization, the profiles are multiplied by 100, 10, 1, and 0.1 from top to bottom.

The time-dependent adsorption of PS-80 at 200 ppm at pH 5.5 is shown in Figure 5a. As indicated previously, the SE measurement started from buffer solution, with the PS-80 solution being injected at the 3rd min. Rapid increase in the adsorbed PS-80 occurred over the subsequent 2–3 min. The adsorption equilibrated with the plateau amount achieved at 1.23 mg/m^2^ after some 15 min. The structure of the adsorbed surfactant layer at equilibrium was determined by neutron reflectivity profiles measured from h-PS-80 in D_2_O, CMCOE-3, and CMAir and d-PS-80 in D_2_O as shown in Figure 5b, with the best model fitted parameters listed in Table S6. The fitted structure parameters are listed in Table 4. Model analysis revealed that the adsorbed surfactants adopted a two-layer structure with no penetration into the PDMS film. The two layers are about 15 Å each, but the 1st layer on the PDMS substrate contains the tails of PS-80 with a volume fraction of 0.3 and the heads of 0.25, while diffuse layer 2 only has the head with a volume fraction of 0.2, as schematically shown in Figure 4c. Thus, the entire adsorbed surfactant layer is 30 Å thick with the tail and head groups rather well separated.

**Table 4:** Best Fitted Structural Parameters for the NR Profiles of PS-80 Adsorption from 200 ppm Solution onto the PDMS Layer

layer	thickness (Å)	Φ_PS-80 tail_	Φ_PS-80 head_	Φ_water_	Γ_PS-80_ (mg/m^2^)
PDMS	50 ± 5	0	0	0.1	0
1	15 ± 3	0.25 ± 0.03	0.30 ± 0.03	0.45 ± 0.03	0.88 ± 0.05
2	15 ± 3	0	0.20 ± 0.03	0.8 ± 0.03	0.32 ± 0.05

#### 3.3.2 Adsorption of COE-3 onto Pre-Adsorbed PS-80

The equilibrated PS-80 layer was rinsed with His buffer. No change to the adsorbed amount was observed, indicating that the adsorbed PS-80 forms a stable layer. 100 ppm COE-3 was subsequently injected, and it can be seen from Figure 5a that there is little change in the total adsorbed amount. As already described previously, some of the adsorbed surfactant molecules might be replaced by COE-3, while the total adsorbed amount remained the same. NR measurements were thus undertaken from the same interface after COE-3 co-adsorption, with the reflectivity profiles shown in Figure S8a. Data analysis indicated that the structure of the adsorbed PS-80 remained unchanged, and the best fitted parameters are shown in Table S7. This confirms no COE-3 co-adsorption onto the PS-80 layer. The result thus informs the inhibiting effect of the pre-adsorbed PS-80 layer on antibody adsorption at the PDMS/water interface. The less affinity of COE-3 to the PDMS surface brings on the incapacity of its replacement of the adsorbed PS-80 on the PDMS layer. This inhibitory interfacial process is schematically summarized in Figure 4d.

### 3.4 Adsorption from the Mixture of COE-3 and PS-80

The dynamic adsorption from the mixed COE-3 and PS-80 solution is shown by the blue squares in Figure 5a, and the structural features of the adsorbed layer were studied by NR with representative reflectivity profiles and best fitted parameters shown in Figure S8b and Table S8, respectively. As evident in Figure 5a, the SE dynamic adsorption from the mixture overlaps with the sequential PS-80-COE-3 adsorption, indicating the competitive adsorption from PS-80 in both cases. The analysis of the NR profiles also confirmed that PS-80 was the only component that was adsorbed with the same structure and composition as the layer formed from the sequential adsorption at the PDMS/water interface. These studies suggest the dominant adsorption of PS-80 even from the pre-mixed solution. The interfacial adsorption from the binary mixture is clearly dominated by diffusion. As the surfactant is much smaller and has a sufficiently high concentration, it can reach the interface and form an adsorbed layer to prevent COE-3 from adsorption.^15^

## 4 Discussion

### 4.1 Adsorption/Desorption of mAbs/PS-80 Affected by Substrate Chemistry and Amphiphilicity

During pre-filled syringe manufacturing, silicone oil is commonly sprayed on the inner barrel of the syringes. When highly concentrated mAb solutions are filled into the pre-filled syringe, the mAb could adsorb onto the silicone oil layer. Additionally, the presence of the surfactant in the antibody formulation could impact the integrity of the oil film. The mAb used in this study, solution conditions, and the PDMS surface have been chosen as a model system to represent interfacial interactions that can occur in mAb combination products using a siliconized syringe. PDMS nanofilms with weak silanization were stable and worked well to facilitate both SE and NR measurements, leading to reproducible results. No change in PDMS film thickness or hydration was observed during the experiments. No penetration of the surfactant or mAb into the PDMS films was detected either, making it easy to focus the data analysis on the mAb–surfactant interactions across the interface. Surfactant or mAb penetration could destabilize the PDMS film. Neither happened based on the studies accumulated, but both COE-3 and PS-80 did adsorb strongly and form a stable layer at the PDMS/water interface, suggesting that while the PDMS surface is hydrophobic, there is no driving force for PS-80, COE-3, or their mixture to penetrate into the PDMS film. The present study has helped clarify the interfacial interactions involving the surfactant.

The most important finding from this work is the formation of a dense 16 Å inner layer and a diffuse 20 Å outer layer upon COE-3 adsorption at 100 ppm, the concentration leading to the saturated adsorption at the PDMS/water interface. Because the shortest axial lengths of Fab, Fc, or the whole COE-3 are about 40–45 Å, the exceedingly thin and dense COE-3 main layer strongly suggests that structural deformation occurred. Further study of the adsorption of Fc and Fab fragments on PDMS confirmed that both Fc and Fab deform when adsorbing on the PDMS surface. The irreversible adsorption of proteins at different interfaces has been widely reported, e.g., the bovine serum albumin (BSA) deformation adsorbed at the air/liquid interface by Noskov et al.^32^ D’Imprima et al. investigated the structures of yeast fatty acid synthase at the air/water interface by electron cryo-tomography and single-particle image processing and found that around 90% of the adsorbed protein complexes at the air/water interface are partly denatured.^33^ Several other studies have also revealed the denaturing and unfolding effects of the self-assembled monolayer (SAM) surfaces on adsorbed proteins such as lysozyme, BSA, and IgG1.^34−36^ As the contact angle of the PDMS film surface is about 95° and is very close to the values measured from hydrocarbon-based SAMs,^37,38^ the results from the PDMS surface could be compared directly. However, SAM surfaces formed from long hydrocarbon chains such as octyltrimethoxysilane (C8) or octadecyltrimethoxysilane (C18) anchored onto silica give a higher adsorbed amount than the PDMS layer, as shown in Figure S9. For different mAb concentrations, the mAb adsorbed amounts on C18 were the highest, while the adsorbed amount on PDMS was the lowest. The low adsorbed amount is often observed from those with short hydrocarbon chains such as methyl, ethyl, and butyl groups with intermediate surface hydrophobicity. Thus, the outer PDMS surface is comparable to methyl terminal groups anchored to the SiO_2_ surface as the two types of surfaces end up with similar protein adsorption. The adsorbed amounts of COE-3 on chlorotrimethylsilane (C1) are shown in Figure S9, displaying a similar low adsorption as that on the PDMS layer when compared to that on the self-assembled C18 and C8 surfaces.

It is also useful to compare the PDMS surface adsorption of COE-3 with that observed at the hydrophilic silica/water interface. The saturated adsorbed amount of the COE-3 layer formed at the PDMS/water interface tends to be 1.49 mg/m^2^ at 100 ppm. This is compared to the saturated adsorbed amount of 2.35 mg/m^2^ achieved at just 10 ppm under the same buffer condition on silica.^24^ The adsorbed layer on SiO_2_ is composed of an inner layer of 40–45 Å and an outer layer of 20 Å, consistent with a predominant flat-on conformation and some tilted projections from the fragments. These observations indicate that the amphiphilic COE-3 has a stronger affinity toward the weakly charged and hydrophilic SiO_2_ surface, but the interaction with the substrate is not strong enough to cause structural deformation or unfolding. Surface hydrophobicity thus has a significant impact on the antibody’s adsorbed amount, orientation, critical saturation points, and loss of its stable structure.

As the deformed COE-3 layer is composed of the main inner dense layer (layer 1) and the outer diffuse layer (layer 2) on the PDMS nanofilm surface, hydrophobic patches within the deformed globular COE-3 framework are likely exposed for PS-80 molecules to bind based on the overlapping distributions of COE-3 and PS-80. Note that Figure 4c,d reveals small but measurable difference in the tail and head distributions of the surfactant, with a distinct skewing of the tail distribution to the PDMS/water interface. This must be caused by hydrophobic interactions between PDMS and COE-3. In addition to the binding of the surfactant molecules to the surface-confined hydrophobic segments, adsorption also occurs on exposed PDMS surface patches.

### 4.2 Adsorption/Desorption of mAbs on Hydrophobic Surfaces in the Presence of Other Nonionic Surfactants

Other surface active species such as PS-20 and poloxamer 188 have also been explored for their roles in inhibiting mAb adsorption.^13,17^ PS-20 is a nonionic surfactant with a similar molecular structure to PS-80, but instead of an oleic acid tail, PS-20 has a lauric acid tail. On the other hand, poloxamer 188 is a nonionic copolymer also possessing strong amphiphilicity. A recent study by Zhang et al.^17^ has investigated the co-adsorption of poloxamer 188 with the mAb at the PDMS/water interface. Poloxamer 188 could prevent the mAb from adsorption when it was injected either before or with the mAb. However, only 16 vol % of the mAb could be removed by the surfactant if the mAb was pre-adsorbed, with the layer thickness for the mAb–surfactant complexes being expanded from 41 to 75 Å. In contrast, our results indicated that no layer expansion occurred following the sequential adsorption of PS-80 and that some 60% of the pre-adsorbed mAb could be removed. The varied mAb removal abilities must arise from their different amphiphilicity implicated by their molecular structures. Oleyl, lauroyl, and the polypropylene oxide block in poloxamer 188 must interact differently with the COE-3 molecules due to their different sizes and chemical nature.

Apart from the chemical structure, surfactant hydrophobicity also played an important role in mAb adsorption/desorption on the hydrophobic surface. For example, PS-20 is generally more hydrophilic than PS-80 due to the shorter alkyl tail. The more hydrophilic PS-20 is also able to prevent a large extent of proteins from adsorption onto the hydrophobic surface at the typical concentrations applied. Desorption of mAb tends to be protein type-dependent. Some proteins such as albumin can be significantly desorbed by PS-20 (up to 90% protein removal),^39^ but a small effect was observed on the desorption of other IgG proteins.^40^ A study by Couston et al. indicated that PS-80 had more impact on mAb desorption at a lower concentration than PS-20 on a silanized surface.^36^ Despite the lack of direct observation of COE-3 desorption by PS-20 from this hydrophobic surface, it is reasonable to expect that PS-80 would have a greater or equal impact on mAb desorption from the hydrophobic surface given that it has a longer hydrophobic tail group. Competitive adsorption phenomena between the protein and surfactant were also observed. Kamalanathan and Martin reported competitive adsorption of Triton X-100 in a model foam fractionation system containing Triton X-100 and BSA, and the displacement of BSA from the surface was attributed to the high surface pressure and diffusivity.^41^ Tein et al. reported that mAb adsorption in surfactant-free solutions creates a monolayer with significant viscoelasticity. This is in contrast to the adsorption of PS20 that leads to an interface with negligible interfacial viscosity. Through competitive adsorption, PS20 can thus protect the air/water interface from mAb adsorption.^13^ When compared to that of the hydrophobic PDMS/liquid interface, the hyperhydrophobicity of the air/liquid interface could also enhance the competitive adsorption of the amphiphilic surfactant to proteins, while the hydrophilic SiO_2_/liquid interface could inhibit the competitive adsorption due to its less affinity to surfactants.^24^

### 4.3 Sequential Injections of mAbs and PS-80

Three different sequences of mAb–surfactant co-adsorption were investigated in this work to examine the role of PS-80 in mitigating mAb adsorption at the silicone oil/water interface. When PS-80 was injected before or with COE-3, PS-80 showed greater surface activity and diffusivity than COE-3, preventing COE-3 from adsorbing on the surface and the subsequent structural deformation. It is very common for mAbs to be formulated with a surfactant (represented by the mixed COE-3 + PS-80 condition). Couston et al. demonstrated that there was little binding between mAb-1 (COE-3) and PS-80, indicating that it is unlikely that PS-80 would prevent COE-3 from adsorption via an intermediate step of complextion.^36^ Pre-adsorption of the surfactant to modify the vessel or container surfaces such as syringes, ampules, or bottles during drug product manufacturing is a less practical approach, but the study condition helps elucidate the role of PS-80 in mitigating protein adsorption.

When the antibody was injected before PS-80, loosely adsorbed mAb molecules were replaced by PS-80 quickly, and the exact amount of removal could be adjusted by controlling the desorption time and surfactant concentration. Given the commonality of this feature, this approach could benefit practical applications such as hormonal assays, biocatalysis, and protein nanoengineering where stable immobilization of bioactive proteins is the key step. For example, the antigen binding efficiency of a concentrated antibody is reduced due to high steric hindrance caused by dense surface packing, as previously shown in a process mimicking pregnancy testing.^42^ A similar trend was observed in the interfacial processes mimicking prostate cancer testing.^43^ However, COE-3 adsorbed onto the PDMS surface suffers from structural deformation and possible loss of bioactivity. These deformed molecules have the risk of being released into the bulk protein solution when the impact of disruption on the surface occurs during the plunger depression during injection. The released protein might induce the aggregation of the bulk protein. It will be useful to check the bioactivity of the mAb adsorbed on PDMS and the impact of surface packing density.

## 5 Conclusions

Although extensive studies were carried out to gain insightful information on the co-adsorption of mAbs and nonionic surfactants on the siliconized surfaces,^17,24,26^ the structural features of the co-adsorbed mAb–surfactant systems remained complex. The current work has helped clarify crucial changes in the interfacial structure and composition. SE measurements have helped outline the main interfacial dynamic features under different adsorption sequences. NR in combination with isotopic labeling provides resolution at the molecular level in determining the structure and composition of COE-3 and PS-80 alone and their co-adsorption. We have revealed that the silanized PDMS nanofilms are stable but contain about 10% of water due to their hydration. Although PS-80 and COE-3 adsorb strongly onto the PDMS surface, the large difference in their surface affinity explains why pre-adsorbed PS-80 and the mixture of PS80/COE-3 can prevent COE-3 from co-adsorbing. However, irreversibly pre-adsorbed COE-3 is heavily deformed, and the unfolded mAb layer attracts PS-80 binding, forming a stable layer of PS-80. In spite of strong interfacial adsorption features, none of the surface-active species penetrated into the PDMS nanofilms which remained highly stable during the experiments. These results thus provide useful information on the role of PS-80 toward mitigating mAb adsorption to the PDMS surface by investigating the interactions involved. Future work will examine how physicochemical properties of other proteins, such as fusion proteins, affect their adsorption at the PDMS/water interface and the roles of different nonionic surfactants in mediating interfacial adsorption and surface-induced aggregation.

## Supporting Information Available

Surface tension data measured from d-PS-80 and h-PS-80, AFM images, contact angle, SE data, neutron reflectivity profiles and best model fits, key parameters from data analysis, and area per molecule and orientations of Fab, Fc, and the whole COE-3 with and without PS-80 at the PDMS/water interface (PDF)

## References

[ref1] Hollowell P.; Li Z.; Hu X.; Ruane S.; Kalonia C.; van der Walle C. F.; Lu J. R. Recent Advances in Studying Interfacial Adsorption of Bioengineered Monoclonal Antibodies. Molecules 2020, 25, 2047 10.3390/molecules25092047. 32353995 PMC7249052

[ref2] Deb P.; Molla M. M. A.; Saif-Ur-Rahman K. M. An Update to Monoclonal Antibody as Therapeutic Option against COVID-19. Biosaf. Health 2021, 3, 87–91. 10.1016/j.bsheal.2021.02.001. 33585808 PMC7872849

[ref3] Jahanshahlu L. Monoclonal antibody as a potential anti-COVID-19. In Biomedicine & Pharmacotherapy, 2020, Vol. 129. 10.1016/j.biopha.2020.110337 PMC7269943 32534226

[ref4] Taylor P. C.; Adams A. C.; Hufford M. M.; de la Torre I.; Winthrop K.; Gottlieb R. L. Neutralizing Monoclonal Antibodies for Treatment of COVID-19. Nat. Rev. Immunol. 2021, 21, 382–393. 10.1038/s41577-021-00542-x. 33875867 PMC8054133

[ref5] Lee H. J.; McAuley A.; Schilke K. F.; McGuire J. Molecular Origins of Surfactant-Mediated Stabilization of Protein Drugs. Adv. Drug Delivery Rev. 2011, 63, 1160–1171. 10.1016/j.addr.2011.06.015. 21763375

[ref6] Pinholt C.; Hartvig R. A.; Medlicott N. J.; Jorgensen L. The Importance of Interfaces in Protein Drug Delivery - Why Is Protein Adsorption of Interest in Pharmaceutical Formulations?. Expert Opin. Drug Delivery 2011, 8, 949–964. 10.1517/17425247.2011.577062. 21557707

[ref7] Spallek M. Method of coating elastomeric components. U.S. Patent US 6123991 A, 2000.

[ref8] Badkar A.; Wolf A.; Bohack L.; Kolhe P. Development of Biotechnology Products in Pre-Filled Syringes: Technical Considerations and Approaches. AAPS PharmSciTech 2011, 12, 564–572. 10.1208/s12249-011-9617-y. 21538214 PMC3134644

[ref9] Chan E.; Hubbard A.; Sane S.; Maa Y. F. Syringe Siliconization Process Investigation and Optimization. PDA J. Pharm. Sci. Technol. 2012, 66, 136–150. 10.5731/pdajpst.2012.00856. 22492599

[ref10] Kerwin B. A. Polysorbates 20 and 80 Used in the Formulation of Protein Biotherapeutics: Structure and Degradation Pathways. J. Pharm. Sci. 2008, 97, 2924–2935. 10.1002/jps.21190. 17973307

[ref11] Smith C.; Li Z.; Holman R.; Pan F.; Campbell R. A.; Campana M.; Li P.; Webster J. R. P.; Bishop S.; Narwal R.; Uddin S.; van der Walle C. F.; Lu J. R. Antibody Adsorption on the Surface of Water Studied by Neutron Reflection. mAbs 2017, 9, 466–475. 10.1080/19420862.2016.1276141. 28353420 PMC5384797

[ref12] Fainerman V. B.; Lotfi M.; Javadi A.; Aksenenko E. V.; Tarasevich Y. I.; Bastani D.; Miller R. Adsorption of Proteins at the Solution/Air Interface Influenced by Added Nonionic Surfactants at Very Low Concentrations for Both Components. 2. Effect of Different Surfactants and Theoretical Model. Langmuir 2014, 30, 12812–12818. 10.1021/la502964y. 25291443

[ref13] Tein Y. S.; Zhang Z.; Wagner N. J. Competitive Surface Activity of Monoclonal Antibodies and Nonionic Surfactants at the Air-Water Interface Determined by Interfacial Rheology and Neutron Reflectometry. Langmuir 2020, 36, 7814–7823. 10.1021/acs.langmuir.0c00797. 32551695

[ref14] Ruane S.; Li Z.; Campana M.; Hu X.; Gong H.; Webster J. R. P.; Uddin F.; Kalonia C.; Bishop S. M.; van der Walle C. F.; Lu J. R. Interfacial Adsorption of a Monoclonal Antibody and Its Fab and Fc Fragments at the Oil/Water Interface. Langmuir 2019, 35, 13543–13552. 10.1021/acs.langmuir.9b02317. 31510747

[ref15] Dixit N.; Maloney K. M.; Kalonia D. S. Protein-Silicone Oil Interactions: Comparative Effect of Nonionic Surfactants on the Interfacial Behavior of a Fusion Protein. Pharm. Res. 2013, 30, 1848–1859. 10.1007/s11095-013-1028-1. 23568525

[ref16] Dixit N.; Maloney K. M.; Kalonia D. S. Application of Quartz Crystal Microbalance to Study the Impact of PH and Ionic Strength on Protein-Silicone Oil Interactions. Int. J. Pharm. 2011, 412, 20–27. 10.1016/j.ijpharm.2011.03.062. 21497645

[ref17] Zhang Z.; Marie Woys A.; Hong K.; Grapentin C.; Khan T. A.; Zarraga I. E.; Wagner N. J.; Liu Y. Adsorption of Non-Ionic Surfactant and Monoclonal Antibody on Siliconized Surface Studied by Neutron Reflectometry. J. Colloid Interface Sci. 2021, 584, 429–438. 10.1016/j.jcis.2020.09.110. 33091867 PMC11165629

[ref18] Campana M.; Webster J. R. P.; Zarbakhsh A. Structural Studies of Nonionic Dodecanol Ethoxylates at the Oil-Water Interface: Effect of Increasing Head Group Size. Langmuir 2014, 30, 10241–10247. 10.1021/la502559r. 25111340

[ref19] Li P. X.; Thomas R. K.; Penfold J. Limitations in the Use of Surface Tension and the Gibbs Equation to Determine Surface Excesses of Cationic Surfactants. Langmuir 2014, 30, 6739–6747. 10.1021/la501287v. 24853780

[ref20] Wolf M. P.; Salieb-Beugelaar G. B.; Hunziker P. PDMS with Designer Functionalities—Properties, Modifications Strategies, and Applications. Prog. Polym. Sci. 2018, 83, 97–134. 10.1016/j.progpolymsci.2018.06.001.

[ref21] Funke S.; Matilainen J.; Nalenz H.; Bechtold-Peters K.; Mahler H. C.; Friess W. Analysis of Thin Baked-on Silicone Layers by FTIR and 3D-Laser Scanning Microscopy. Eur. J. Pharm. Biopharm. 2015, 96, 304–313. 10.1016/j.ejpb.2015.08.009. 26316044

[ref22] Lu J. R.; Thomas R. K. Neutron Reflection from Wet Interfaces. J. Chem. Soc., Faraday Trans. 1998, 94, 995–1018. 10.1039/a707853f.

[ref23] Lu J. R.; Zhao X.; Yaseen M. Protein Adsorption Studied by Neutron Reflection. Curr. Opin. Colloid Interface Sci. 2007, 12, 9–16. 10.1016/j.cocis.2007.02.001.

[ref24] Li Z.; Pan F.; Li R.; Pambou E.; Hu X.; Ruane S.; Ciumac D.; Li P.; Welbourn R. J. L.; Webster J. R. P.; Bishop S. M.; Narwal R.; van der Walle C. F.; Lu J. R. Coadsorption of a Monoclonal Antibody and Nonionic Surfactant at the SiO 2 /Water Interface. ACS Appl. Mater. Interfaces 2018, 10, 44257–44266. 10.1021/acsami.8b16832. 30500160

[ref25] Li Z.; Li R.; Smith C.; Pan F.; Campana M.; Webster J. R. P.; van der Walle C. F.; Uddin S.; Bishop S. M.; Narwal R.; Warwicker J.; Lu J. R. Neutron Reflection Study of Surface Adsorption of Fc, Fab, and the Whole MAb. ACS Appl. Mater. Interfaces 2017, 9, 23202–23211. 10.1021/acsami.7b06131. 28613817

[ref26] Pan F.; Li Z.; Leyshon T.; Rouse D.; Li R.; Smith C.; Campana M.; Webster J. R. P.; Bishop S. M.; Narwal R.; van der Walle C. F.; Warwicker J.; Lu J. R. Interfacial Adsorption of Monoclonal Antibody COE-3 at the Solid/Water Interface. ACS Appl. Mater. Interfaces 2018, 10, 1306–1316. 10.1021/acsami.7b13332. 29215260

[ref27] Hu X.; Pambou E.; Gong H.; Liao M.; Hollowell P.; Liu H.; Wang W.; Bawn C.; Cooper J.; Campana M.; Ma K.; Li P.; Webster J. R. P.; Padia F.; Bell G.; Lu J. R. How Does Substrate Hydrophobicity Affect the Morphological Features of Reconstituted Wax Films and Their Interactions with Nonionic Surfactant and Pesticide?. J. Colloid Interface Sci. 2020, 575, 245–253. 10.1016/j.jcis.2020.04.043. 32361410

[ref28] De Feijter J. A.; Benjamins J.; Veer F. A. Ellipsometry as a Tool to Study the Adsorption Behavior of Synthetic and Biopolymers at the Air–Water Interface. Biopolymers 1978, 17, 1759–1772. 10.1002/bip.1978.360170711.

[ref29] Cubitt R.; Fragneto G. D17: The New Reflectometer at the ILL. Appl. Phys. A: Mater. Sci. Process. 2002, 74, S329–S331. 10.1007/s003390201611.

[ref30] Pambou E.; Li Z.; Campana M.; Hughes A.; Clifton L.; Gutfreund P.; Foundling J.; Bell G.; Lu J. R. Structural Features of Reconstituted Wheat Wax Films. J. R. Soc., Interface 2016, 13, 20160396 10.1098/rsif.2016.0396. 27466439 PMC4971226

[ref31] Nelson A. Co-Refinement of Multiple-Contrast Neutron/X-Ray Reflectivity Data Using MOTOFIT. J. Appl. Crystallogr. 2006, 39, 273–276. 10.1107/S0021889806005073.

[ref32] Noskov B. A.; Mikhailovskaya A. A.; Lin S. Y.; Loglio G.; Miller R. Bovine Serum Albumin Unfolding at the Air/Water Interface as Studied by Dilational Surface Rheology. Langmuir 2010, 26, 17225–17231. 10.1021/la103360h. 20961051

[ref33] D’Imprima E.; Floris D.; Joppe M.; Sánchez R.; Grininger M.; Kühlbrandt W. Protein Denaturation at the Air-Water Interface and How to Prevent It. Elife 2019, 8, 1–18. 10.7554/eLife.42747. PMC6443348 30932812

[ref34] Lu J. R.; Su T. J.; Thirtle P. N.; Thomas R. K.; Rennie A. R.; Cubitt R. The Denaturation of Lysozyme Layers Adsorbed at the Hydrophobic Soild/Liquid Surface Studied by Neutron Reflection. J. Colloid Interface Sci. 1998, 206, 212–223. 10.1006/jcis.1998.5680. 9761646

[ref35] Shouren Ge S.; Kojio K.; Takahara A.; Kajiyama T. Bovine Serum Albumin Adsorption onto Immobilized Organotrichlorosilane Surface: Influence of the Phase Separation on Protein Adsorption Patterns. J. Biomater. Sci., Polym. Ed. 1998, 9, 131–150. 10.1163/156856298X00479. 9493841

[ref36] Couston R. G.; Skoda M. W.; Uddin S.; van der Walle C. F. Adsorption Behavior of a Human Monoclonal Antibody at Hydrophilic and Hydrophobic Surfaces. mAbs 2013, 5, 126–139. 10.4161/mabs.22522. 23196810 PMC3564877

[ref37] Wang J.; Jia D.; Tao K.; Wang C.; Zhao X.; Yaseen M.; Xu H.; Que G.; Webster J. R. P.; Lu J. R. Interfacial Assembly of Lipopeptide Surfactants on Octyltrimethoxysilane- Modified Silica Surface. Soft Matter 2013, 9, 9684–9691. 10.1039/c3sm51271a. 25692456

[ref38] Jin J.; Wang L.; Zheng Z.; Zhang J.; Hu X.; Lu J. R.; Etor D.; Pearson C.; Song A.; Wood D.; Gallant A. J.; Balocco C. Metal-Insulator-Metal Diodes Based on Alkyltrichlorosilane Self-Assembled Monolayers. AIP Adv. 2019, 9, 065017 10.1063/1.5100252.

[ref39] Zhang M.; Ferrari M. Reduction of Albumin Adsorption onto Silicon Surfaces by Tween 20. Biotechnol. Bioeng. 1997, 56, 618–625. 10.1002/(SICI)1097-0290(19971220)56:6<618::AID-BIT4>3.0.CO;2-Q. 18642333

[ref40] Minhua Feng M.; Berdugo Morales A. B.; Poot A.; Beugeling T.; Bantjes A. Effects of Tween 20 on the Desorption of Proteins from Polymer Surfaces. J. Biomater. Sci., Polym. Ed. 1996, 7, 415–424. 10.1163/156856295X00427. 8562519

[ref41] Kamalanathan I. D.; Martin P. J. Competitive Adsorption of Surfactant-Protein Mixtures in a Continuous Stripping Mode Foam Fractionation Column. Chem. Eng. Sci. 2016, 146, 291–301. 10.1016/j.ces.2016.03.002.

[ref42] Cowsill B. J.; Waigh T. A.; Eapen S.; Davies R.; Lu J. R. Interfacial Structure and History Dependent Activity of Immobilised Antibodies in Model Pregnancy Tests. Soft Matter 2012, 8, 9847–9854. 10.1039/c2sm26133b.

[ref43] Zhao X.; Pan F.; Garcia-Gancedo L.; Flewitt A. J.; Ashley G. M.; Luo J.; Lu J. R. Interfacial Recognition of Human Prostate-Specific Antigen by Immobilized Monoclonal Antibody: Effects of Solution Conditions and Surface Chemistry. J. R. Soc., Interface 2012, 9, 2457–2467. 10.1098/rsif.2012.0148. 22552922 PMC3427501

