# Supplementary material for: Competitive Adsorption of a Monoclonal Antibody and Nonionic Surfactant at the PDMS/Water Interface
Source: Mol Pharm. 2023 Apr 4;20(5):2502–12. doi: 10.1021/acs.molpharmaceut.2c01099 (PMC10155179; doi:10.1021/acs.molpharmaceut.2c01099)
Supplement: Supplementary file 1 — mp2c01099_si_001.pdf [file mp2c01099_si_001.pdf]

Supporting information

Competitive adsorption of monoclonal antibody and nonionic surfactant at the PDMS/water interface

Kangcheng Shen[1], Xuzhi Hu[1]*, Zongyi Li[1], Mingrui Liao[1] Zeyuan Zhuang[1], Sean Ruane[1], Ziwei Wang[2], Peixun Li[3], Samantha Micciulla[4], Kasinathan Narayanan[5], Cavan Kalonia[6], Jian Ren Lu[1]*

1 Biological Physics Laboratory, School of Physics and Astronomy, University of Manchester, Oxford Road, Schuster Building, Manchester M13 9PL, U.K.

2 National Graphene Institute, University of Manchester, Oxford Road, Schuster Building, Manchester M13 9PL, U.K.

3 STFC ISIS Facility, Rutherford Appleton Laboratory, Didcot, OX11 0QX, UK.

4 Institut Laue-Langevin, 71 Avenue des Martyrs, CS-20156, 38042 Grenoble, France

5 Dosage Form Design & Development, AstraZeneca, Granta Park, Cambridge CB21 6GH, U.K.

6 Dosage Form Design & Development, AstraZeneca, Gaithersburg, Maryland 20878, United States

* E-mail: j.lu@manchester.ac.uk Tel: (+44)-161-200-3926.

Keywords: mAb, interface, desorption, adsorption, polysorbate, neutron reflection, polydimethylsiloxane, spectroscopic ellipsometry

Section S1 Spectroscopic Ellipsometry (SE)

A common SE measurement procedure is as follow: the unpolarized light is converted to linearly polarized and then incident on the sample surface, converting to the elliptically polarized light and being detected. The ratio of complex Fresnel reflection coefficients, ρ, can be expressed as shown in equation S1. R_P and R_S are the Fresnel coefficients of s and p waves after reflection. ρ can be parametrized by the phase shift Δ and the amplitude variation Ψ when the polarized light is reflected by the sample[1].

$$\rho = \frac{R_p}{R_s} = \tan(\Psi)e^{i\Delta} \qquad \text{(S1)}$$

The Ellipsometer M-2000U was purchased from J.A.Woollam Co. Inc and the data was analyzed using CompleteEASE. The analysis procedure applies a theoretical model which contains the information of layer thickness and optical constants. The experimental and theoretical data are compared regressively until the best match of both is found. The accuracy of this estimation process can be quantified by the mean square error (MSE). The layer thickness d and the refractive index n, which are coupled, can be calculated by this procedure. To determine the unique d, n is first calculated using the Cauchy's equation

$$n(\lambda) = A + \frac{B}{\lambda^2} \qquad \text{(S2)}$$

where λ is the wavelength, A and B are the Cauchy's coefficients. In this experiment, A and B are taken as 1.45 and 0.003 for both protein and surfactants, 1.4 and 0 for PDMS layer, respectively. The surface adsorbed amount, Γ, is calculated using the De Feijter's equation[2].

$$\Gamma = d\frac{(n - n_0)}{dn/dc} \qquad \text{(S3)}$$

where n and n_0 are the refractive indices of the sample layer and the ambient

environment, dn/dc is the refractive index increment of the sample and is taken as 0.18 mL/g for protein and 0.12 mL/g for surfactant[3,4]. A common issue of SE is the coupling of layer thickness and refractive index for an ultrathin layer (<15 nm), but the coupled n and d can lead to the estimate of the adsorbed amount by using equation (S3) without the need to decouple thickness from refractive index.

To check the thickness of the silicon oxide layer on the silicon wafer/block we take the refractive index of the pure SiO_2 layer without any pore and defect. For the wafers and polished silicon blocks, the native oxide layers usually are 14±3Å thick and the absence of any defects has been confirmed by neutron reflection.

The dynamic measurement of the protein adsorption process was undertaken under "in situ" mode in which situation the silicon wafer was assembled in a purposely built SE liquid cell with a pair of fused quartz windows with the incident and exiting angles fixed to 70°.

Section S2 Neutron Reflection (NR)

NR experiments were undertaken on D17 reflectometer[5] at Institut Laue Langevin laboratory, Grenoble, France. The PDMS modified silicon block was assembled in a liquid cell to facilitate NR measurement at the solid/liquid interface. An alignment process was undertaken to make sure that the beam could be exactly reflected by the sample surface and detected. A transmission process was made to normalize the NR data. NR experiments can unravel the interior structure of the sample layer by recording the neutron reflectivity R, which is the ratio of the intensity of incident/reflected beam, and is plotted against the momentum transfer Q where Q can be expressed as

$$Q = \frac{4\pi\sin\theta}{\lambda} \qquad \text{(S4)}$$

where λ is the beam wavelength and θ is the incident angle. The multi-contrast measurements of NR can be achieved by changing the ratio of H_2O against D_2O in the solvent, which can tune the SLD of the bulk solution. Due to the hydrogen/deuterium

exchange the SLD of protein will vary in different solvent contrasts. The SLD values of all the components are shown in Table S1. The average SLD of a multi-component system can be calculated as follows

$$SLD_{average} = \sum_i S_i \emptyset_i \tag{S5}$$

where S_i and $\emptyset_i$ are the SLD and volume fraction of the ith component. In this experiment, the solvent contrasts are chosen as D_2O (SLD=6.35×10^{-6}Å^{-2}), Null Reflecting Water (NRW, SLD=0) and contrast matched COE-3 (CMCOE-3, SLD=2.58×10^{-6}Å^{-2}). The software used for the NR data analysis was Motofit[6], which enables the construction of the multilayer model containing a series of correlated variables such as layer thickness and average SLD. By using least-square approximation, the experimental reflectivity profile is compared with the calculated profile and the process is iterated to get the best fitted result. By solving equation S5 the volume fractions of each layer components can be determined. With the layer thickness and volume fraction of each component the multilayer model can be built to describe the inner structure of the system. The surface adsorbed amount Γ can be calculated by

$$\Gamma = d\emptyset_i\sigma \tag{S6}$$

where σ is the density of material. The consistency and reliability of Γ can be checked by comparing the NR result to the SE result.

Section S3 Supporting figures and tables for this experiment

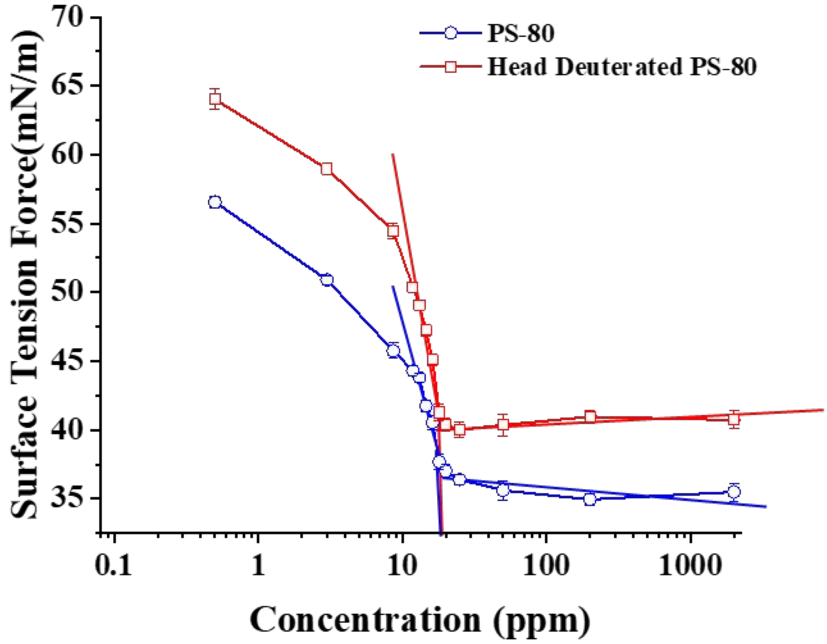

Figure S1: The plot of surface tension measured versus PS-80 (blue) and head-deuterated PS-80 (red) concentrations at the air/water interface. For better visualization, the surface tension force profile of head-deuterated PS-80 is multiplied by 1.1. The CMCs of PS-80 and head-deuterated PS-80 are determined to be around 16 ppm and 17 ppm, respectively.

**Material**	**D_2O**	**PDMS**	**Si**	**SiO_2**	**CMAir**	**CMCOE-3**
**SLD (× 10^{-6} Å^{-2})**	6.35	0.06	2.07	3.47	0	2.58
**Material**	**COE-3 in CMAir**	**COE-3 in D_2O**	**h-PS-80 head**	**h-PS-80 tail**	**d-PS-80 head**	**d-PS-80 tail**
**SLD (× 10^{-6} Å^{-2})**	2.05	3.36	0.65	-0.39	6.7	-0.39

Table S1: SLDs of all materials used in this experiment.

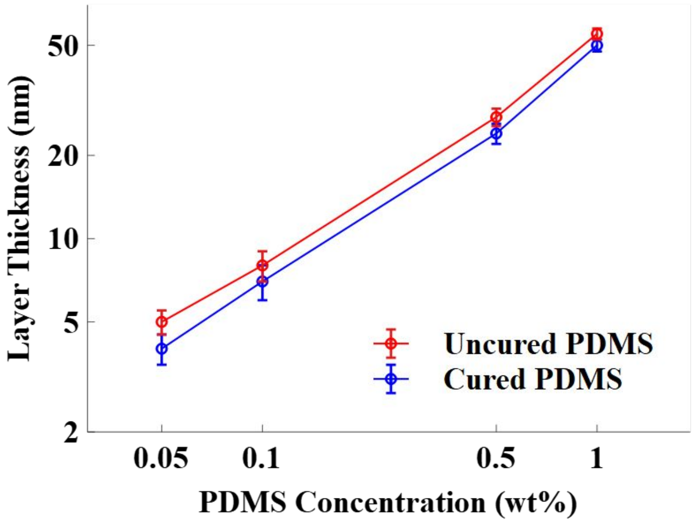

Figure S2: The thickness of cured (blue line) and uncured (red line) PDMS layers on silicon wafer against PDMS concentration (wt% in Hexane). The figure was plotted using the log-log style for a clear view.

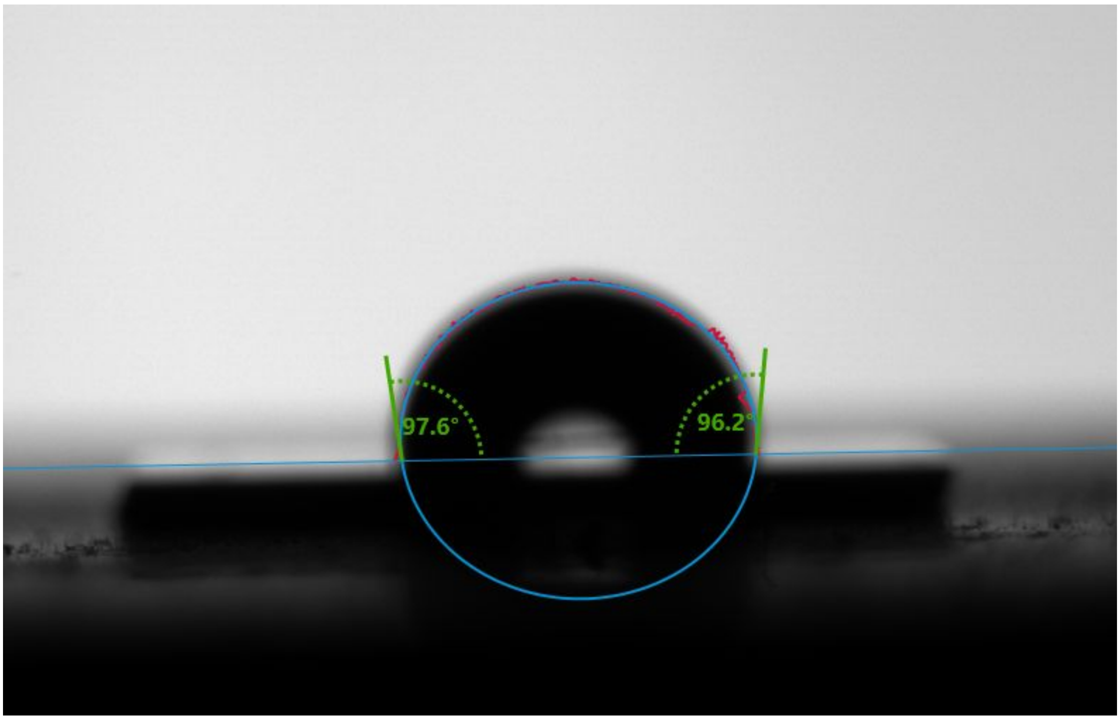

Figure S3: Contact angle measurement from the PDMS coated silicon wafer surface. The average advancing contact angle was found to be 95° ± 5° for all PDMS films coated.

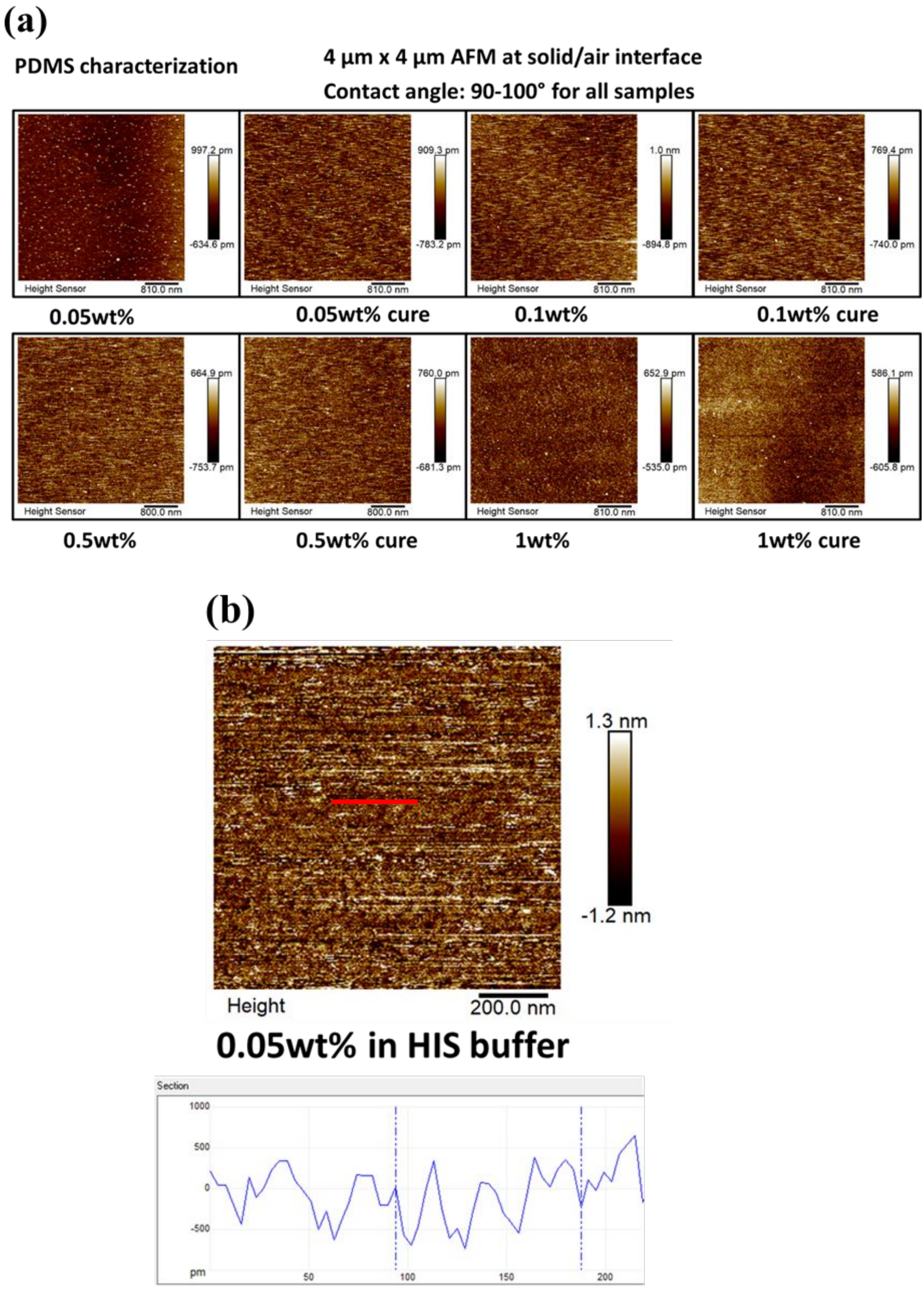

Figure S4: AFM images taken from PDMS films coated at different PDMS concentrations with/without curing at (a) PDMS/air (b) PDMS/water interfaces with height analysis.

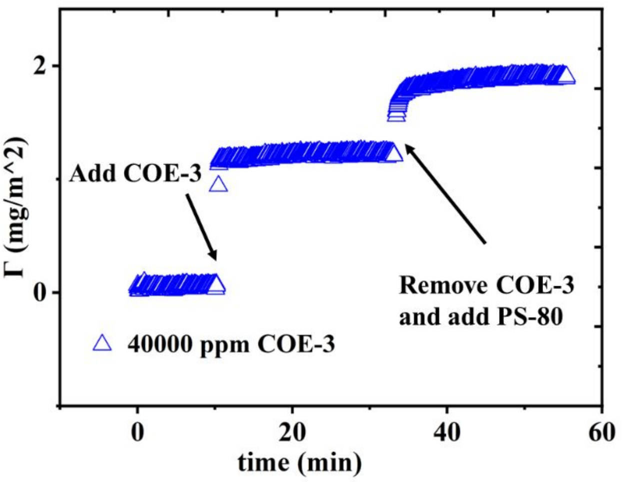

Figure S5: Ellipsometric measurements of adsorption of COE-3 at 40000 ppm, followed by 200 ppm PS-80 at pH5.5, 25mM histidine buffer

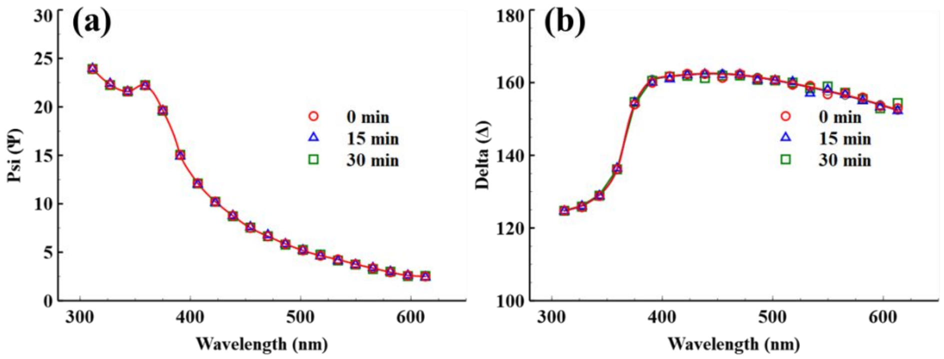

Figure S6: Ellipsometric scans of (a) the amplitude component Ψ and (b) the phase difference Δ against wavelength measured at the PDMS/water interface in His buffer (pH 5.5 with ionic strength of 25mM) in 0, 15 and 30 min. The continuous lines show the uniform layer fits with PDMS layer (50 Å, volume fraction 90%).

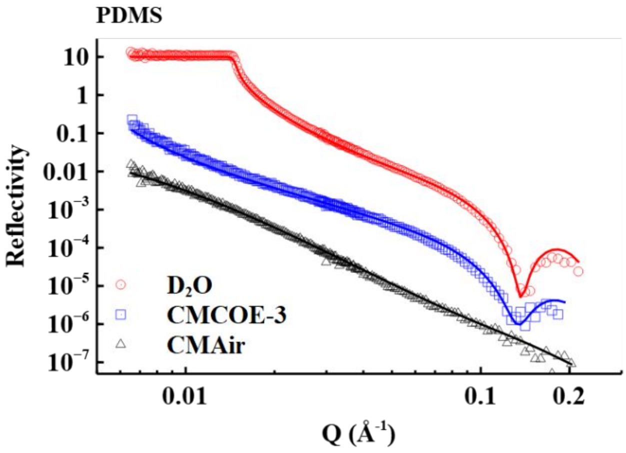

Figure S7: The NR profiles and fitting results of the PDMS film coated on silicon block. For better visualization, the profiles are multiplied by 10, 1 and 0.1 from top to bottom. The best fitted parameters are shown in **Table S2**.

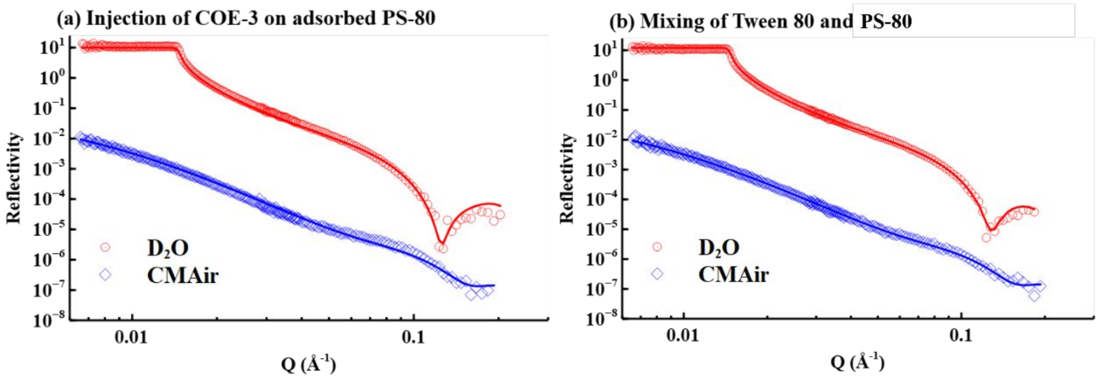

Figure S8: NR profiles measured at the PDMS/water interface from (a) injection of COE-3 on pre-adsorbed h-PS-80layer, (b) the adsorption from the binary mixture of h-PS-80and COE-3. For better visualization, the profiles are multiplied by 10 and 0.1 from top to bottom. The best fitted parameters are shown in **Table S7** and **S8**.

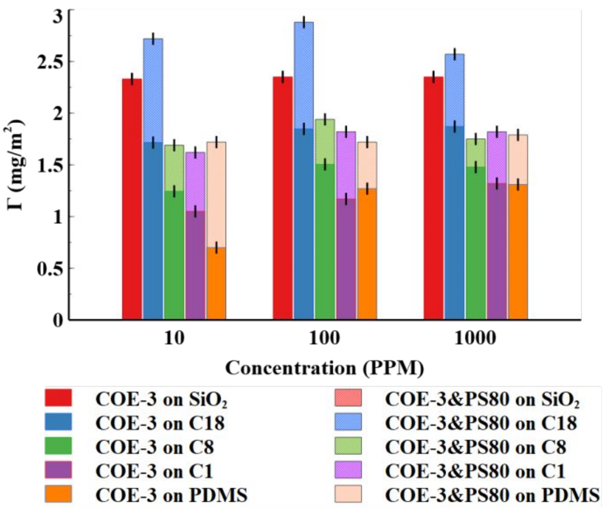

Figure S9: The adsorbed amount of COE-3 at concentrations of 10, 100 and 1000 ppm (in His buffer, pH 5.5, ionic strength of 25mM) onto the SiO2 surface coated with a self-assembled monolayer of hydrophobic C18, C8, C1, PDMS and bare SiO_2 layer, respectively. The results were measured directly from the solid/water interface.

Section S4 Best-fitted parameters for NR profiles

Contrast	Layer	Thickness (Å)	SLD ($\times 10^{-6}$Å^{-2})	Solvent	Roughness (Å)
**D_2O**	SiO_2	10 ± 1	3.47	0.24	0.5
	PDMS	50 ± 5	0.06	0.1	0.5
**CMCOE-3**	SiO_2	10 ± 1	3.47	0.24	0.5
	PDMS	50 ± 5	0.06	0.1	0.5
**CMAir**	SiO_2	10 ± 1	3.47	0.24	0.5
	PDMS	50 ± 5	0.06	0.1	0.5

Table S2: Best-fit parameters of Figure S5 in three isotopic contrasts: D_2O, CMCOE-3 and CMAir. Solvent represents hydration for layer SiO_2 and PDMS.

Contrast	Layer	Thickness (Å)	SLD ($\times 10^{-6}$Å^{-2})	Solvent	Roughness (Å)
**D_2O**	SiO_2	10 ± 1	3.47	0.24	0.5
	PDMS	50 ± 5	0.06	0.1	0.5
	1	16 ± 2	4.64	0	0.5
	2	20 ± 2	6.01	0	3
**CMCOE-3**	SiO_2	10 ± 1	3.47	0.24	0.5
	PDMS	50 ± 5	0.06	0.1	0.5
	1	16 ± 2	2.63	0	0.5
	2	20 ± 2	2.63	0	3
**CMAir**	SiO_2	10 ± 1	3.47	0.24	0.5
	PDMS	50 ± 5	0.06	0.1	0.5
	1	16 ± 2	0.92	0	3
	2	20 ± 2	0.22	0	12

Table S3: Best-fit parameters of Figure 1 (b) for the two-layer model in three isotopic contrasts: D_2O, CMCOE-3 and CMAir. Solvent represents hydration for layer SiO_2 and PDMS. The solvent hydration for layer 1 and 2 was fixed as 0. The volume fractions of surfactant, mAb and solvent were calculated from SLD values obtained from different contrasts in layer 1 and 2.

Material	Layer	Thickness (Å)	SLD ($\times 10^{-6}$Å^{-2})	Solvent	Roughness (Å)
**Fc**	SiO_2	12 ± 1	3.47	0.24	0.5
	PDMS	59 ± 5	0.06	0.13	0.5
	1	16 ± 2	4.90	0	0.5
	2	20 ± 2	6.00	0	3
**Fab**	SiO_2	10 ± 1	3.47	0.15	0.5
	PDMS	58 ± 5	0.06	0.15	0.5
	1	16 ± 2	4.97	0	0.5
	2	20 ± 2	6.10	0	3

Table S4: Best-fit parameters of **Figure 2** for the two-layer model in D_2O. Solvent represents hydration for layer SiO2 and PDMS. The solvent hydration for layer 1 and 2 was fixed as 0. The volume fractions of surfactant, mAb and solvent were calculated from SLD values obtained from different contrasts in layer 1 and 2.

Contrast	Layer	Thickness (Å)	SLD ($\times 10^{-6}$Å^{-2})	Solvent	Roughness (Å)
**D_2O (d-PS-80)**	SiO_2	10 ± 1	3.47	0.24	0.5
	PDMS	50 ± 5	0.06	0.1	0.5
	1	16 ± 2	4.41	0	0.5
	2	20 ± 2	6.29	0	3
**CMCOE-3 (d-PS-80)**	SiO_2	10 ± 1	3.47	0.24	0.5
	PDMS	50 ± 5	0.06	0.1	0.5
	1	16 ± 2	3.12	0	0.5
	2	20 ± 2	3.39	0	3
**CMAir (d-PS-80)**	SiO_2	10 ± 1	3.47	0.24	0.5
	PDMS	45 ± 5	0.06	0.1	0.5
	1	16 ± 2	1.32	0	0.5
	2	20 ± 2	0.91	0	3
**D_2O (h-PS-80)**	SiO_2	10 ± 1	3.47	0.24	0.5
	PDMS	50 ± 5	0.06	0.1	0.5
	1	16 ± 2	3.26	0	0.5
	2	20 ± 2	5.15	0	3
**CMCOE-3(h-PS-80)**	SiO_2	10 ± 1	3.47	0.24	0.5
	PDMS	50 ± 5	0.06	0.1	0.5
	1	16 ± 2	1.50	0	0.5
	2	20 ± 2	2.29	0	3
**CMAir (h-PS-80)**	SiO_2	10 ± 1	3.47	0.24	0.5
	PDMS	50 ± 5	0.06	0.1	0.5
	1	16 ± 2	0.45	0	0.5

	2	20 ± 2	0.28	0	3

Table S5: Best-fit parameters to the reflectivity profiles shown in Figures 3 (a) and (b) for the two-layer model involving h-Tween and d-Tween in three isotopic contrasts: D_2O, CMCOE-3 and CMAir.

Contrast	Layer	Thickness (Å)	SLD ($\times10^{-6}$Å^{-2})	Solvent	Roughness (Å)
**D_2O (h-PS-80)**	SiO_2	10 ± 1	3.47	0.24	0.5
	PDMS	50 ± 5	0.06	0.1	0.5
	1	15 ± 3	2.92	0	0.5
	2	15 ± 3	5.68	0	3
**CMCOE-3(h-PS-80)**	SiO_2	10 ± 1	3.47	0.24	0.5
	PDMS	50 ± 5	0.06	0.1	0.5
	1	15 ± 3	1.51	0	0.5
	2	15 ± 3	2.40	0	3
**CMAir (h-PS-80)**	SiO_2	10 ± 1	3.47	0.24	0.5
	PDMS	50 ± 5	0.06	0.1	0.5
	1	15 ± 3	0.35	0	0.5
	2	15 ± 3	0.07	0	3
**D_2O (d-PS-80)**	SiO_2	10 ± 1	3.47	0.24	0.5
	PDMS	40 ± 5	0.06	0.1	0.5
	1	15 ± 3	3.71	0	0.5
	2	15 ± 3	6.51	0	3

Table S6: Best-fit parameters to the reflectivity profiles shown in Figure 5 (b) for the two-layer model fits involving h-PS-80 in three solvent isotopic contrasts: D_2O, CMCOE-3 and CMAir and d-PS-80 in D_2O.

Contrast	Layer	Thickness (Å)	SLD ($\times10^{-6}$Å^{-2})	Solvent	Roughness (Å)
**D_2O (h-PS-80)**	SiO_2	12 ± 1	3.47	0.24	0.5
	PDMS	50 ± 5	0.06	0.1	0.5
	1	15 ± 3	2.85	0	0.5
	2	15 ± 3	5.72	0	3
**CMAir (h-PS-80)**	SiO_2	12 ± 1	3.47	0.24	0.5
	PDMS	50 ± 5	0.06	0.1	0.5
	1	15 ± 3	0.51	0	0.5
	2	15 ± 3	0.10	0	3

Table S7: Best-fit parameters to the reflectivity profiles shown in Figure S6 (a) for the two-layer model fits of COE-3 adsorption on pre-adsorbed h-PS-80 in D_2O and CMAir.

**Contrast**	Layer	Thickness (Å)	SLD ($\times10^{-6}$Å^{-2})	Solvent	Roughness (Å)
**D_2O (h-PS-80)**	SiO_2	12 ± 1	3.47	0.24	0.5
	PDMS	50 ± 5	0.06	0.1	0.5

	1	15 ± 3	2.78	0	0.5
	2	15 ± 3	5.78	0	3
**CMAir (h-PS-80)**	SiO_2	12 ± 1	3.47	0.24	0.5
	PDMS	50 ± 5	0.06	0.1	0.5
	1	15 ± 3	0.32	0	0.5
	2	15 ± 3	0.09	0	3

Table S8: Best-fit parameters to the reflectivity profiles shown in Figure S6 (b) for the two-layer model fits involving the mixture of h-PS-80 and COE-3 in D_2O and CMAir.

References

(1) Pan, F.; Li, Z.; Leyshon, T.; Rouse, D.; Li, R.; Smith, C.; Campana, M.; Webster, J. R. P.; Bishop, S. M.; Narwal, R.; Van Der Walle, C. F.; Warwicker, J.; Lu, J. R. Interfacial Adsorption of Monoclonal Antibody COE-3 at the Solid/Water Interface. *ACS Appl. Mater. Interfaces* **2018**, *10* (1), 1306–1316. https://doi.org/10.1021/acsami.7b13332.

(2) De Feijter, J. A.; Benjamins, J.; Veer, F. A. Ellipsometry as a Tool to Study the Adsorption Behavior of Synthetic and Biopolymers at the Air–Water Interface. *Biopolymers* **1978**, *17* (7), 1759–1772. https://doi.org/10.1002/bip.1978.360170711.

(3) Zhao, X.; Pan, F.; Cowsill, B.; Lu, J. R.; Garcia-Gancedo, L.; Flewitt, A. J.; Ashley, G. M.; Luo, J. Interfacial Immobilization of Monoclonal Antibody and Detection of Human Prostate-Specific Antigen. *Langmuir* **2011**, *27* (12), 7654–7662. https://doi.org/10.1021/la201245q.

(4) Li, Z.; Pan, F.; Li, R.; Pambou, E.; Hu, X.; Ruane, S.; Ciumac, D.; Li, P.; Welbourn, R. J. L.; Webster, J. R. P.; Bishop, S. M.; Narwal, R.; Van Der Walle, C. F.; Lu, J. R. Coadsorption of a Monoclonal Antibody and Nonionic Surfactant at the SiO 2 /Water Interface. *ACS Appl. Mater. Interfaces* **2018**, *10* (51), 44257–44266. https://doi.org/10.1021/acsami.8b16832.

(5) Cubitt, R.; Fragnetto, G. D17: The New Reflectometer at the ILL. *Appl. Phys. A Mater. Sci. Process.* **2002**, *74* (1), S329–S331. https://doi.org/10.1007/s003390201611.

(6) Nelson, A. Co-Refinement of Multiple-Contrast Neutron/X-Ray Reflectivity Data Using MOTOFIT. *J. Appl. Crystallogr.* **2006**, *39* (2), 273–276. https://doi.org/10.1107/S0021889806005073.
